# Using event-related potentials to track morphosyntactic development in second language learners: The processing of number and gender agreement in Spanish

**DOI:** 10.1371/journal.pone.0200791

**Published:** 2018-07-27

**Authors:** José Alemán Bañón, Robert Fiorentino, Alison Gabriele

**Affiliations:** 1 Centre for Research on Bilingualism, Department of Swedish and Multilingualism, Stockholm University, Stockholm, Sweden; 2 Neurolinguistics & Language Processing Laboratory, Department of Linguistics, University of Kansas, Lawrence, Kansas, United States of America; 3 Second Language Acquisition Laboratory, Department of Linguistics, University of Kansas, Lawrence, Kansas, United States of America; Penn State University, UNITED STATES

## Abstract

We used event-related potentials to investigate morphosyntactic development in 78 adult English-speaking learners of Spanish as a second language (L2) across the proficiency spectrum. We examined how development is modulated by the similarity between the native language (L1) and the L2, by comparing number (a feature present in English) and gender agreement (novel feature). We also investigated how development is impacted by structural distance, manipulating the distance between the agreeing elements by probing both within-phrase (*fruta* *muy* *jugosa* “fruit_-FEM-SG_ very juicy_-FEM-SG_”) and across-phrase agreement (*fresa* *es* *ácida* “strawberry_-FEM-SG_ is tart_-FEM-SG_”). Regression analyses revealed that the learners’ overall proficiency, as measured by a standardized test, predicted their accuracy with the target properties in the grammaticality judgment task (GJT), but did not predict P600 magnitude to the violations. However, a relationship emerged between immersion in Spanish-speaking countries and P600 magnitude for gender. Our results also revealed a correlation between accuracy in the GJT and P600 magnitude, suggesting that behavioral sensitivity to the target property predicts neurophysiological sensitivity. Subsequent group analyses revealed that the highest-proficiency learners showed equally robust P600 effects for number and gender. This group also elicited more positive waveforms for within- than across-phrase agreement overall, similar to the native controls. The lowest-proficiency learners showed a P600 for number overall, but no effects for gender. Unlike the highest-proficiency learners, they also showed no sensitivity to structural distance, suggesting that sensitivity to such linguistic factors develops over time. Overall, these results suggest an important role for proficiency in morphosyntactic development, although differences emerged between behavioral and electrophysiological measures. While L2 proficiency predicted behavioral sensitivity to agreement, development with respect to the neurocognitive mechanisms recruited in processing only emerged when comparing the two extremes of the proficiency spectrum. Importantly, while both L1-L2 similarity and hierarchical structure impact development, they do not constrain it.

## Introduction

The present study uses event-related potentials “ERPs” to investigate morphosyntactic development in a group of 78 adult English-speaking learners of Spanish as a second language (L2) at three levels of proficiency (low, intermediate, high). The study focuses on inflectional morphology, a domain of grammar that is known to be problematic for adult L2 learners across the proficiency spectrum (e.g. [1–6]). More specifically, we examine noun-adjective number and gender agreement. An example of these agreement relations in Spanish is shown in (1), where the noun *manzanas* “apples” triggers number and gender agreement on both the attributive adjective *rojas* “red”, which is located within the determiner phrase (DP), and the predicative adjective *deliciosas* “delicious”, which is located across a verb phrase (VP):

$$\begin{array}{l} {}_{\mathrm{DP}}[\mathrm{Las}\;\underline{\mathrm{manzanas}}\;\underline{\mathrm{rojas}}]\;{}_{\mathrm{VP}}[\mathrm{son}\;\underline{\mathrm{deliciosas}}].\\ \;{\mathrm{the}}_{‑\mathrm{FEM}‑\mathrm{PL}}\;{\mathrm{apple}}_{‑\mathrm{FEM}‑\mathrm{PL}}\;{\mathrm{red}}_{‑\mathrm{FEM}‑\mathrm{PL}}\;\mathrm{are}\;{\mathrm{delicious}}_{‑\mathrm{FEM}‑\mathrm{PL}}\\ “\mathrm{Red}\;\mathrm{apples}\;\mathrm{are}\;\mathrm{delicious}.” \end{array}$$ (1)

One of the main questions that we investigate herein concerns how development is impacted by the properties of the learners’ native language (L1). Crucially, the linguistic properties that we focus on differ with respect to their status in our learners’ L1. English realizes number (although not on adjectives), but it does not instantiate grammatical gender. Our study is also among the first to investigate how morphosyntactic development is affected by linguistic factors such as the structural distance between the elements in the agreement dependency (i.e. whether the noun and the adjective belong in the same phrase or in different phrases) (see also [3]).

The question of how L2 grammatical knowledge develops over time has occupied a central role in L2 acquisition research from a variety of frameworks (e.g. [7–23]). Different L2 theoretical models have capitalized on distinct aspects of grammatical development (e.g. acquisition orders/sequences, L1 transfer, ultimate attainment, processing). For example, within the generative tradition, research has mainly focused on (1) the extent to which L2 grammatical development is mediated by the properties of the learners’ L1 and (2) whether development is guided by the same domain-specific mechanisms that are hypothesized to guide L1 acquisition (i.e. Universal Grammar). This tradition is well represented by theoretical models such as the Interpretability Hypothesis [17] and the Full Transfer Full Access Hypothesis [14, 15]. Both models assume that adult L2 learners rely on the properties of their L1 from the start of the acquisition process, but differ with respect to whether or not the development of novel L2 syntactic properties is predicted to be possible. Under the Interpretability Hypothesis, development with respect to novel L2 uninterpretable features (e.g. gender on adjectives) is not predicted to be possible, due to a permanent representational deficit at the level of the syntax (see also [24, 25]). In contrast, under the Full Transfer Full Access Hypothesis, there may be facilitation for properties that are instantiated in the L1 at lower levels of proficiency but, with increasing proficiency, development of all grammatical categories of the L2 to native-like levels is predicted to be possible (although not guaranteed), regardless of the L1.

While the above proposals are mainly concerned with the development of abstract L2 syntactic knowledge, others have capitalized on the development of the processing procedures or neurocognitive mechanisms involved in L2 morphosyntactic processing (e.g. [9–13, 16, 20–22, 26]). Of particular interest herein are proposals from the ERP literature. ERPs are voltage changes in the electroencephalogram measured at the scalp and time-locked to specific events of interest. As they provide high temporal resolution (with millisecond precision), they have been used to investigate the temporal dynamics of L1 and L2 comprehension. Importantly, different ERPs are modulated by different aspects of language processing, which has shed light on the qualitative nature of both L1 and L2 processing. For example, the P600 is a positive deflection between ~500-1000ms that is typically captured by central-posterior electrodes of the EEG cap (e.g. [27, 28]), and which has been linked to various aspects of morphosyntactic processing (e.g. [27, 29]). In fact, the P600 is the component that is most consistently found for agreement violations in native speakers (see [30] for a review), although there is also evidence that the P600 is not exclusively morphosyntactic in nature, since it can be found for certain types of semantic anomalies (e.g. [31–34]). In some studies, the P600 is preceded by a Left Anterior Negativity (LAN), a negative deflection between ~300-500ms in left anterior areas that is assumed to index automatic morphosyntactic processing (e.g. [30, 35, 36]). A caveat with the LAN, however, is that several studies on morphosyntactic processing do not find it (e.g. [37–40]).

In contrast, the N400 [41] is a negative-going wave between ~300-500ms with a central-posterior distribution, which has been linked to processes of semantic integration and lexical access (see [42, 43] for reviews). Although agreement violations rarely yield an N400 in native speakers (cf. [44] found an N400 for agreement errors in non-sentential contexts; see also [45]), adult L2 learners sometimes show an N400 for agreement violations for which native speakers show a P600 (e.g. [26, 46–48]). Evidence has also been provided for a qualitative shift in L2 processing (i.e. from N400 to P600) as a function of development. Two studies are particularly relevant in this respect. Osterhout et al. [49] tracked morphosyntactic development in English-speaking beginners of French longitudinally (after one, four, and eight months of French classes). They examined number agreement (1) between the subject and the verb (tu adores/*adorez “you_-2nd-SG_ adore_-2nd-SG_/*adore_-2nd-PL_”), which is a syntactic context where English realizes number, and (2) between the noun and the preceding indefinite article (des hamburgers/*hamburger “some_-PL_ hamburguer_-PL_/*hamburger_-SG_”), a context where English does not realize any type of agreement. Violations of both agreement types yielded a P600 in French native speakers. After one month of instruction, the seven L2ers with the highest accuracy in the judgment task showed an N400 for subject-verb violations, an effect which shifted into a P600 after four months. Determiner-noun errors showed no effects at any point. The N400 for subject-verb errors after one month of instruction suggests that learners do not engage in grammatical processing from the start, not even for structures that are similar in the L1 and L2 (contra the transfer models from the generative tradition). However, these results also show that L1-L2 similarity modulates grammatical development to some extent, since even after eight months of instruction the L2ers only showed sensitivity to the property that is similar in English and French.

A subsequent cross-sectional study from the same lab by Tanner et al. [50] examined the development of subject-verb agreement in two groups of English-speaking learners of German, those who progressed through their third year of university German instruction and beginners in their first year. In L1-German controls, agreement errors (e.g. Ich wohne/*wohnt in Berlin “I live_-1st-SG_/*live_-3rd-SG_ in Berlin) elicited a P600. This was also the pattern for third-year learners. In contrast, the first-year students showed a biphasic N400-P600 pattern, caused by some learners showing an N400 and others, a P600. Tanner et al. argue for discrete L2 learning stages. At an earlier stage, processing mainly relies on lexical-semantic mechanisms and, therefore, morphosyntactic violations yield an N400. Later on, processing relies on the application of rule-based grammatical knowledge, and agreement violations yield a P600. The biphasic N400-P600 pattern in first-year students suggests that learners are variable in how fast they transition from one stage to the next.

This idea of a qualitative shift in L2 morphosyntactic development is well represented by theoretical proposals such as Ullman’s Declarative/Procedural Model [20–22]. Ullman posits that, at lower levels of proficiency, the neural bases and neurocognitive mechanisms associated with L1 and L2 morphosyntactic processing are qualitatively distinct, although development is assumed to be possible. Initially, learners use lexical-semantic information (subserved by declarative memory and indexed by the N400) for automatic rule-based operations that are associated with procedural memory in native speakers (e.g. phrase structure, morphosyntax). However, as proficiency and experience with the L2 increase, there is a gradual shift from reliance on declarative to procedural memory and L2 morphosyntactic processing becomes increasingly qualitatively native-like (i.e. LAN-P600). It is implied that this developmental trajectory applies to all morphosyntactic properties of the L2, regardless of L1-L2 similarity (consistent with Osterhout et al. [49] and Tanner et al. [50]) and other linguistic factors (e.g. structural distance), although the model is not specific about the role of grammar-internal factors. This was largely the pattern reported by Morgan-Short et al. [51], who examined the processing of gender agreement in an artificial language (Brocanto2) by L1-English learners. At a low level of proficiency, gender violations were mainly associated with N400 effects. At a high level of proficiency, the N400 shifted into a P600, but only for noun-determiner gender violations. Noun-adjective gender violations still elicited an N400. Thus, these results also show that certain factors that are internal to the grammar being acquired (i.e. syntactic configuration) impact development. Our study contributes to this line of research by examining how development is impacted by structural distance, a question that remains largely unexplored.

Along similar lines, Steinhauer et al. [16] propose six stages of development in L2 morphosyntactic processing. In Stage 1, novice learners show no sensitivity to the target property. In Stage 2 (very low-proficiency), sensitivity emerges, but morphosyntactic dependencies are processed at the lexical level and, thus, violations yield N400 effects, as opposed to a P600. In Stages 3-4 (low/intermediate), L2ers undergo a qualitative shift and begin to compute morphosyntactic dependencies as such; this is when the P600 emerges and consolidates. Finally, in Stages 5-6 (advanced/near-native), L2 processing becomes increasingly native-like, and anterior negativities emerge (at least, for computations for which L1 speakers also show them).

Not every proposal from the L2 processing literature assumes that development is possible, though. The Shallow Structure Hypothesis [52, 53] posits that adult L2 learners have a permanent deficit at the level of the syntax and, therefore, they cannot build fine-grained syntactic representations online, regardless of proficiency and regardless of the properties of their L1 (e.g. [54]). Unlike Ullman [20–22] and Steinhauer et al. [16], the advocates of the Shallow Structure Hypothesis claim that native-like processing in adult L2ers is restricted to local relationships, such as within-phrase agreement. For nonlocal morphosyntactic dependencies, it is assumed that L2ers resort to the use of lexical-semantic or pragmatic strategies. Thus, development of qualitatively native-like L2 processing routines is not predicted to go beyond local relationships under this model.

In sum, the above proposals differ along a number of dimensions. Only proposals from the behavioral literature (Interpretability Hypothesis, Full Transfer Full Access Hypothesis) assign a privileged role to the L1 in the development of L2 grammars, although these models differ with respect to whether or not development for novel L2 syntactic properties is predicted to be possible (at least, in a native-like manner). With respect to the L2 processing accounts, all proposals (Shallow Structure Hypothesis, Declarative/Procedural Model, Steinhauer et al. [16]) assume a certain reliance on lexical-semantic/pragmatic information for all morphosyntactic computations at lower levels of proficiency (unlike the transfer accounts), but they differ with respect to whether or not native-like morphosyntactic processing at higher levels of proficiency is possible. Only Ullman’s Declarative/Procedural model and Steinhauer et al. [16] assume that, with increased proficiency and experience, L2 morphosyntactic processing can become qualitatively native-like for all L2 properties (see also [55]). In contrast, the Shallow Structure Hypothesis specifically predicts that the development of the procedures required for the processing of across-phrase dependencies is not possible for adult L2ers.

The present ERP study investigates L2 morphosyntactic development with an emphasis on the above issues. First, we investigate the qualitative nature of morphosyntactic processing at different stages of development, by examining L2 learners across the proficiency spectrum (low, intermediate, high). In addition, we examine the extent to which development is mediated by L1-L2 similarity, by examining a property that is present in the learners’ L1 (number) and one that is unique to their L2 (gender). Importantly, we also examine how linguistic factors such as structural distance impact the development of sensitivity to morphosyntactic dependencies, by comparing within-phrase and across-phrase agreement (i.e. local vs. nonlocal). Within this section, we provide a succinct description of the relevant linguistic properties (number and gender) in Spanish, we summarize the most relevant literature on morphosyntactic development, and we introduce our own study and research questions.

### Number and gender in Spanish

Spanish classifies nouns as masculine or feminine. Thus, learners must be able to assign nouns to their appropriate gender classes as a preliminary step to compute gender agreement. One potential difficulty is that, for entities without biological gender, this lexical classification is largely arbitrary. In addition, neither of the two genders in Spanish is associated with a unique marker. That said, ~99.8% of nouns ending in–*o* are masculine and 96.3% of nouns ending in–*a* are feminine [56]. Thus, these two markers provide strong distributional cues to gender, especially as they make up two thirds of the Spanish lexicon [57]. Since the present study is mainly concerned with how the ability to establish morphosyntactic dependencies develops over time, rather than with lexical assignment, these “transparent” nouns are the focus of the present study.

With respect to number, Spanish has a dual distinction between singular and plural. Singular is morphologically unmarked and plural is formed by adding–*s* or–*es* to the root (e.g. *manzana/manzanas* “apple/apples”, *caracol/caracoles* “snail/snails”) [58]. Here we focus on nouns/adjectives that are pluralized by adding–*s*.

### Literature review

A number of studies have examined L2 morphosyntactic development by comparing learners at different levels of proficiency (e.g. [3, 59–62]). The results of these studies suggest that, although development is possible for all morphosyntactic properties, L2 processing might be less efficient due to L1 transfer. A few ERP studies have also examined the development of the neuro-cognitive mechanisms subserving L2 morphosyntax (alongside the abovementioned studies by Osterhout et al. [49], Tanner et al. [50], and Morgan-Short et al. [51]). For example, Rossi et al. [63] examined subject-verb agreement in L1-German L2-Italian and L1-Italian L2-German learners, with high or low proficiency. Both high-proficiency groups showed a biphasic pattern (LAN-P600), similar to the native controls [64]. Both low-proficiency groups showed a reduced and delayed P600, and no LAN. Interestingly, there was no effect of L1 background, possibly because both German and Italian instantiate subject-verb agreement. These results suggest that the mechanisms recruited for morphosyntactic processing become native-like with increased proficiency. The reader might wonder why the N400-P600 pattern reported for low-proficiency learners in Tanner et al. [50] was not found in Rossi et al.’s study. One possibility is that the low-proficiency learners in Rossi et al.’s study might have been past the lexical-semantic processing stage (they had received two to three years of L2 instruction).

An important point about the studies by Rossi et al. [63] and Tanner et al. [50] is that subject-verb agreement is instantiated in the learners’ L1s. This might explain why even the low-proficiency learners in both studies showed some sensitivity to the violations and why the high-proficiency learners were completely native-like. Other ERP studies have investigated how development is impacted by L1-L2 similarity. For example, Ojima et al. [65] probed subject-verb agreement in L1-Japanese L2-English learners at high and low levels of proficiency. Crucially, Japanese does not realize subject-verb agreement. In English native speakers, violations (e.g. foreigners eat/*eats sushi) elicited a LAN-P600 biphasic pattern. The high-proficiency group showed a LAN but no P600, and the low-proficiency group showed no effects. That only the high-proficiency learners showed a LAN suggests that certain aspects of agreement processing develop with proficiency. However, the absence of a P600 in the high-proficiency group is surprising, since no other L2 study on agreement has reported LAN effects in the absence of a P600 (although Mancini et al. [66] show a similar pattern for person violations in Spanish native speakers). One possibility is that the processes of syntactic reanalysis and repair associated with the P600 might be more likely to be initiated for computations that exist in the L1. However, a review of the L2 literature suggests that, at a high level of proficiency, P600 effects are usually found for novel agreement types (e.g. [48, 67–70]). Another possibility is that the P600 was delayed (as in Rossi et al. [63]) and not captured in the epoch.

White et al. [71] tracked morphosyntactic development in low-proficiency L1-Chinese and L1-Korean learners of English enrolled in a nine-week intensive English course. The study focused on tense verbal inflection (she did not start/*started…; she had not started/*start…). Crucially, Chinese lacks tense inflection. Korean, in contrast, does inflect verbs for tense, but differently from English. The native controls [72] showed a biphasic LAN-P600 pattern for tense errors. The L2ers showed no effects after only one week. However, after eight/nine weeks, tense violations elicited a P600 in both learner groups. Results also revealed a positive correlation between the learners’ accuracy in the GJT and the magnitude of the P600, suggesting that, as behavioral sensitivity to the target property increases, L2 processing recruits (at least partially) similar neurocognitive mechanisms to those involved in native processing (i.e. P600) [49–50]. These results suggest that proficiency plays a more important role in development than L1-L2 similarity.

Finally, Caffarra et al. [73] used logistic regression to examine the unique contribution of L1-L2 similarity, L2 proficiency, immersion in L2-speaking countries, and age of acquisition to L2 syntactic processing (including, but not limited to, morphosyntax). In a meta-analysis of 41 published ERP studies, proficiency emerged as the strongest predictor of the presence of a P600 for syntactic violations. Their analysis, however, did not tease apart global proficiency (as measured by a standardized test) from behavioral accuracy with the target property. Caffarra et al.’s meta-analysis on longitudinal/training studies also revealed that length of instruction was the most reliable predictor of the presence of a P600 for syntactic violations. Length of immersion in L2-speaking countries emerged as the most reliable predictor of the presence of the LAN for syntactic errors (>5 years), although Caffarra et al. point out that other factors (which were not tested) play a role in whether or not the mechanisms indexed by the LAN are in place in L2ers (e.g. working memory). We examine the unique contribution of these factors (overall L2 proficiency, amount of instruction, and length of immersion in L2 speaking countries) in our study.

To our knowledge, our study is among the first to examine how development is impacted by structural distance. Previous ERP studies manipulating structural distance have only examined advanced L2 learners. For example, Gillon-Dowens et al. [69, 70] found that highly proficient L1-English L2-Spanish learners elicited a LAN-P600 pattern for number and gender violations realized within the DP (similar to the native controls). However, violations realized across the VP only elicited a P600 (unlike the controls). Likewise, Foucart & Frenck-Mestre [48] found that advanced L1-English L2-French learners elicited a P600 for within-phrase gender errors, but were insensitive to gender errors realized across the VP. Keating [3] used eye-tracking to examine Spanish gender agreement in L1-English L2-Spanish learners at different stages of development and found that only the advanced learners showed sensitivity to gender, and only in local contexts (within the DP), suggesting that development might be constrained by hierarchical structure (in line with Clahsen & Felser [52, 53]). Importantly, linear and structural distance correlated in all of these studies (i.e. the agreeing words were adjacent in the within-phrase conditions, but separated by one word in the across-phrase conditions), suggesting that the unique contribution of structural distance to agreement processing remains an open question. In previous studies we have investigated the unique contribution of hierarchical structure to the processing of number and gender agreement in both native speakers of Spanish [37] and L1-English L2-Spanish learners [67, 74]. Our design compares within-phrase (*fruta* *muy* *jugosa* “fruit_-FEM-SG_ very juicy_-FEM-SG_”) and across-phrase agreement (*fresa* *es* *ácida* “strawberry_-FEM-SG_ is tart_-FEM-SG_”) while controlling for both linear distance (one word) and for the syntactic category of the agreeing elements (noun-adjective), a factor that was also not teased apart in Gillon-Dowens et al.’s studies [69, 70].

#### Summary and methodological challenges

Overall, a review of the previous literature reveals a strong role for proficiency in the development of the neurocognitive mechanisms associated with L2 morphosyntactic processing. How L1-L2 similarity shapes development is less clear-cut, although the available evidence suggests that it does modulate development. Importantly, no previous ERP study has examined how other linguistic factors, such as structural distance, impact development, although some evidence has been provided that the complexity of the syntactic domain matters (e.g. [51]).

An important observation is that proficiency has been operationalized differently in the above studies. For example, the cross-sectional studies by Rossi et al. [63] and Ojima et al. [65] used a number of instruments to assess L2 proficiency, including performance in a test (translation task, standardized test), self-rated proficiency, amount of L2 instruction, and time spent in L2-speaking countries. In contrast, the cross-sectional study by Tanner et al. [50] segregated learners according to their course level at the time of testing. Likewise, the longitudinal studies by Osterhout et al. [49] and White et al. [71] operationalized proficiency as behavioral accuracy with the target structures, rather than overall L2 proficiency, and these two types of proficiency were not teased apart by Caffarra et al. [73]. In addition, as pointed out by Bowden et al. [55], no previous ERP study has examined the unique contribution of both overall L2 proficiency and factors related to experience with the L2, such as the amount of L2 instruction and length of immersion in L2-speaking countries, with the same group of learners. One of the main contributions of the present study is that we examine the role of both overall L2 proficiency (via performance on a comprehensive test of L2 grammar) and sensitivity to the target structures (via D-Prime scores in the GJT) on morphosyntactic development. Our study is also among the first to evaluate the unique contribution of experiential variables, such as amount of L2 instruction and length of immersion in Spanish-speaking countries, to morphosyntactic development.

### Present study and research questions

The present study tracks L2 morphosyntactic development in a group of adult English-speaking learners of Spanish at different levels of proficiency. The study is one of the largest-scale ERP studies on L2 processing to date, including a total of 78 learners across the proficiency spectrum. As discussed above, we have reported subsets of these data in previous work [67, 74], but the current paper represents the only time that the full data set collected for this multi-year project was analyzed and reported. Thus, all analyses reported herein are original.

The specific research questions (RQ) that inform our study are presented below:

RQ1. How does proficiency modulate morphosyntactic processing in a late-acquired L2? What proficiency measures are the most reliable predictors of sensitivity to morphosyntactic dependencies?

Of particular interest is whether L2 processing undergoes a qualitative change as a function of development, as predicted by models from the L2 processing literature (e.g. Declarative/Procedural Model; Steinhauer et al. [16]). With respect to the role of different proficiency measures, we examine performance on a comprehensive L2 grammar test, length of immersion in L2-speaking countries, and amount of instruction in the L2. In addition, we examine the role of accuracy with the target properties via D-Prime scores in a GJT, to examine the extent to which behavioral sensitivity to the target properties results in electrophysiological sensitivity.

RQ2. To what extent does the similarity between the L1 and L2 impact morphosyntactic development?

We address this question by comparing number agreement, which is instantiated in the learners’ L1, and gender agreement, which is unique to the L2. Both agreement types will be examined on adjectives, a syntactic context where English does not instantiate any type of agreement. As the above literature review suggests, novel features (i.e. gender) and different instantiations of shared features (i.e. number on adjectives) are the types of properties for which we can expect to see development across the proficiency spectrum. Recall that, under the transfer models from the generative tradition (Full Transfer Full Access Hypothesis, Interpretability Hypothesis), we might find evidence for grammatical processing for number at lower levels of proficiency (i.e. P600). In addition, only the Full Transfer Full Access Hypothesis predicts that development is possible for gender (at least, to native-like levels). In contrast, proposals from the processing literature (e.g. Declarative/Procedural Model; Steinhauer et al. [16]) assume a certain reliance on lexical semantic mechanisms (i.e. N400) for all morphosyntactic dependencies at lower levels of proficiency, even for properties that exist in the L1. With the exception of the Shallow Structure Hypothesis, these proposals also assume that development is possible as a function of proficiency and experience with the L2.

RQ3. How does structural distance (i.e. hierarchical structure) modulate the development of sensitivity to morphosyntactic dependencies in the L2?

We address this question by probing agreement both when the noun and the adjective are located within the same phrase and when they are located across phrases. Among the processing proposals, the Declarative/Procedural Model and Steinhauer et al. [16] do not assume a specific role for linguistic factors such as structural distance in development, but recall that the complexity of the syntactic context was found to impact development in Morgan-Short et al.’s study [51]. In contrast, under the Shallow Structure Hypothesis, development is not predicted to be possible for across-phrase dependencies. To our knowledge, this is the first ERP study that investigates how linguistic factors such as structural distance impact the development of sensitivity to agreement dependencies.

Our design manipulates structural distance by probing both within-phrase (*fruta* *muy* *jugosa* “fruit_-FEM-SG_ very juicy_-FEM-SG_”) and across-phrase agreement (*fresa* *es* *ácida* “strawberry_-FEM-SG_ is tart_-FEM-SG_”) while controlling both for linear distance and for the syntactic category of the agreeing elements. For both Spanish native speakers [37] and advanced learners [67], our results revealed robust P600 effects for both number and gender violations, both within and across the phrase. The P600 was larger for number than gender, but planned comparisons revealed that this effect was driven by the advanced L2ers. Importantly, our results also showed more positive waveforms for all within-phrase conditions relative to their across-phrase counterparts between 400-900ms in both groups, similar to previous studies which have examined the role of hierarchical structure on agreement processing (e.g. [75, 76]). We interpreted these results as evidence that both native speakers and advanced learners posit hierarchical syntactic representations online. The same design will be employed herein to examine how morphosyntactic development is impacted by hierarchical structure.

## Materials and methods

The project was approved by the Human Subjects Committee at the University of Kansas (#17510). Participants provided their written informed consent to participate in this study.

### Participants

In total, 81 L2 learners of Spanish (55 females) participated in the study after providing their informed consent. Data from 25 advanced learners are reported in Alemán Bañón et al. [67]. A preliminary report including data from 11 intermediate and 11 low-proficiency learners can be found in Gabriele et al. [74]. The learners were all native speakers of English with no significant exposure to Spanish or any other languages before puberty. Data from three learners (3 females) were removed from analyses due to an excessive amount of artifacts in the recordings. The learners’ proficiency was measured with the reading/vocabulary section of the MLA Cooperative Language Test (Spanish Embassy, Washington, DC, USA) and the cloze test from the Diploma de Español como Lengua Extranjera (Educational Testing Service, Princeton, NJ, USA). This is a 50-item test which has been used in previous studies on the acquisition of number and gender agreement by L2 learners of Spanish (e.g. [2, 4, 59, 61, 68, 77, 78]). Table 1 provides information regarding all 78 learners’ scores in the proficiency test (“Proficiency Test Score”), amount of Spanish instruction (“Instruction”), and length of immersion in Spanish-speaking countries (“Months Abroad”). Information regarding the learners’ age of acquisition and age at the time of testing is also provided.

**Table 1:** Proficiency and age information about the L2 learners (ungrouped data; *N* = 78).

Proficiency Test Score	range: 12-50; mean: 33.51; SD: 10.32
Instruction (years)	range: 0-9; mean: 3.14; SD: 1.50
Months Abroad	range: 0-30; mean: 6.69; SD: 8.50
Age at Testing	range: 19-41; mean: 23; SD: 4
Age of Acquisition	range: 10-22; mean: 14.27; SD: 2.82

Instruction: Number of years of L2 instruction; Months Abroad: Number of Months immersed in an L2-speaking country

The 24 native speakers of Spanish (13 females) reported in Alemán Bañón et al. [37] served as the control group, for comparison. All of the native speakers indicated having being exposed to Spanish from birth, having been raised in a Spanish-speaking country (Spain, Bolivia, or Paraguay) until at least age 17, and having being schooled in Spanish. They were all L2 learners of English, but they all reported using Spanish on a daily basis. All 78 learners reported here were right-handed, as assessed by the Edinburgh Handedness Inventory [79], had normal or corrected to normal vision, and indicated no history of neurological or linguistic disabilities. Most of them were students at a major university in the US, where all of them were tested, and they all received $10 per hour of participation.

### Materials

The materials were the same as those used in Alemán Bañón et al. [37, 67]. They include 120 sentences which manipulate agreement within the Determiner Phrase (DP) and 120 sentences which manipulate agreement across a Verb Phrase (VP). A sample of the stimuli is provided in Table 2. The agreeing words are underlined.

**Table 2:** Sample stimuli for the experimental conditions.

**Within-Phrase Agreement**
**Grammatical** 1. La manzana es una fruta muy jugosa y la papaya también. the apple is _**DP**_[a fruit_-FEM-SG_ very juicy_-FEM-SG_] and the papaya too.
**Number Violation** 2. La manzana es una fruta muy *jugosas y la papaya también. the apple is _**DP**_[a fruit_-FEM-SG_ very juicy_-FEM-PL_] and the papaya too.
**Gender Violation** 3. La manzana es una fruta muy *jugoso y la papaya también. the apple is _**DP**_[a fruit_-FEM-SG_ very juicy_-MASC-SG_] and the papaya too.
**Across-Phrase Agreement**
**Grammatical** 4. La fresa es ácida y la piña también. the strawberry_-FEM-SG **VP**_[is tart_-FEM-SG_] and the pineapple too.
**Number Violation** 5. La fresa es *ácidas y la piña también. the strawberry_-FEM-SG **VP**_[is tart_-FEM-PL_] and the pineapple too.
**Gender Violation** 6. La fresa es *ácido y la piña también. the strawberry_-FEM-SG **VP**_[is tart_-MASC-SG_] and the pineapple too.

FEM: Feminine; MASC: Masculine; PL: Plural; SG: Singular; DP: Determiner Phrase; VP: Verb Phrase; “*”: agreement violation

As shown in Table 2, agreement in the two contexts (within-phrase, across-phrase) is realized between a noun and an adjective. In both configurations, violations become noticeable on the adjective (a syntactic context where English does not instantiate any type of agreement). In the within-phrase conditions, the agreeing elements are located within a Determiner Phrase (DP). Therefore, the parser does not cross any phrase boundaries when checking agreement between the adjective and the noun (_DP_[*fruta* *muy* *jugosa*] “fruit very juicy”). In contrast, in the across-phrase conditions, the noun and the adjective are located across a Verb Phrase (VP). Thus, their agreement relation can only be checked once the parser has crossed the verb phrase (*fresa*_VP_[*es* *ácida*] “strawberry is tart”). In both contexts, the linear distance between the agreeing elements was one word (the adverb *muy* “very” in the within-phrase conditions; the copula “be” inflected for third person singular present tense *es* in the across-phrase condition).

Only singular nouns were used in the study. In order to avoid attraction errors (e.g. [80, 81]) there were no intervening nouns between the agreeing elements in any of the experimental conditions. All adjectives formed the plural by suffixing–*s* to the stem, as in *jugosa/jugosas*, “juicy”. This ensured that participants received overt cues to resolve number agreement, a factor which has been found to impact number resolution in native speakers (e.g. [30, 82]). Within each context (within-phrase, across-phrase), we used an equal number of masculine and feminine nouns (the same is true of the whole study), all of which referred to inanimate entities such that learners could not rely on biological gender to establish syntactic gender agreement. All nouns and adjectives in the stimuli were canonically marked for gender (masculine–*o* and feminine–*a*) (e.g. [40, 44, 69, 70, 83–85].

We used the LEXESP database to calculate the mean log frequency of the nouns and adjectives where we examined agreement (via BuscaPalabras, [86]). Both the nouns and adjectives in the within-phrase and across-phrase conditions were controlled for both frequency and length (adjective frequency: *t*(238) = -.678, *p* = .45; adjective length: *t*(238) = -.414, *p* = .68; noun frequency: *t*(238) = -.841, *p* = .40; noun length: *t*(238) = -.546, *p* = .58). In addition, all critical nouns and adjectives were used twice. However, since the testing involved two separate EEG recordings (see *Procedure* below), stimulus lists were created such that, at a given recording, participants would only see one version of each critical adjective, in order to minimize potential repetition effects on the waveforms.

An additional 120 sentences from a separate experiment that examines agreement between demonstratives and nouns, but which does not manipulate structural distance were included in the study (reported in Alemán Bañón et al. (e.g. [67]). Furthermore, given that a large proportion of ungrammatical sentences in the overall design runs the risk of attenuating the P600 (e.g. [87, 88]), we added another 120 grammatical fillers to the experiment. This brought the ratio of grammatical to ungrammatical stimuli to 1:1. Overall, each participant saw 40 sentences from each of the six experimental conditions in Table 2 (80 grammatical sentences, 80 number violations, and 80 gender violations), 120 sentences from another experiment (40 grammatical sentences, 40 number violations, and 40 gender violations), and the 120 grammatical fillers. Three presentation lists were created using a Latin Square design, such that participants only saw one version of each experimental sentence per list.

### Procedure

#### EEG recording and behavioral tasks

The testing was divided into two sessions (e.g. [68, 84]), separated by a minimum of two days and a maximum of two weeks. Participants received instructions to silently read a series of Spanish sentences and judge whether they were grammatically correct or not (e.g. [50, 51, 63, 71]). They were asked to favor accuracy over speed and to avoid blinks and body movements while reading the sentences. Each recording began with a practice set of nine sentences, five of which were ungrammatical. None of the practice items included agreement errors, and none included nouns or adjectives from the experimental stimuli. Participants received feedback for the first three practice trials, to ensure they understood the task. The experiment began immediately after the practice. Each recording consisted of six blocks of 40 sentences, separated by short breaks. Sentences were presented one word at a time using the RSVP (Rapid Serial Visual Presentation) method. Within each block, sentences from all experimental conditions were intermixed and randomized. Words were displayed in black text (Courier New font) against a grey background. All Spanish words showed the appropriate diacritics and the last word of each sentence was marked with a period. The presentation of the stimuli was carried out using *Paradigm* by Perception Research Systems, Inc. [89].

Each trial began with a fixation cross, which was displayed for 500ms. Immediately after, the first word of the sentence was presented. Each word was displayed for 450ms, followed by a 300ms blank screen (e.g. [37, 68, 90]). At the end of each sentence, there was a 1000ms pause, after which the participants saw the prompts for the GJT (*Bien* for grammatical sentences and *Mal* for ungrammatical ones). The prompts remained on the screen until the participant provided a response. Responses were provided with the left hand: middle finger for correct sentences and index finger for incorrect ones. Following the behavioral response, there was an interval between trials ranging from 500-1000ms, pseudorandomly varied at 50ms increments.

After the second recording, learners took two additional tasks. One of them was a computerized task testing knowledge of lexical gender. Learners were presented with 180 nouns, which includes all trigger nouns in the within-phrase and across-phrase conditions, and asked to select the definite article with which they agreed: *La* (feminine singular) or *El* (masculine singular). The second task was a vocabulary task. Learners read 110 Spanish words and were asked to circle the correct English translation from among two options. The task included 20 nouns and 20 adjectives from the within-phrase conditions and 20 nouns and 20 adjectives from the across-phrase conditions. All learners performed very well in both tasks.

### EEG recording

The EEG was continuously recorded by means of 32 sintered Ag/AgCl scalp electrodes attached to an elastic cap (Electro-Cap International, Inc.) and placed in a modified 10-20 layout (midline: FPZ, FZ, FCZ, CZ, CPZ, PZ, OZ; lateral: FP1/2, F7/8, F3/4, FT7/8, FC3/4, T3/4, C3/4, TP7/6, CP3/4, T5/6, P3/4, O1/2). Electrode AFZ served as the ground electrode. The physically linked mastoids were used as reference during the recording. Horizontal eye movements were measured with two electrodes on the left and right outer canthi. An additional four electrodes above and below each eye were used to monitor blinks. Impedance was maintained below 5 kΩ for scalp electrodes, and below 10kΩ for eye electrodes. The recordings were amplified by a Neuroscan Synamps2 amplifier (Compumedics Neuroscan, Inc.) with a bandpass filter of 0.1 to 200 Hz, and digitized continuously with a sampling rate of 1 kHz.

We used the Neuroscan Edit software (Compumedics Neuroscan, Inc.) and EEGLAB to analyze the EEG data. Trials with artifacts (e.g. blinks, horizontal eye movements, excessive muscle movement, or excessive alpha waves) were manually rejected before analysis. A minimum of 20 trials per condition was established for a given learner to be included in the analyses. Approximately the same number of trials was preserved per condition (range: 20 to 38 out of 40). The rest of the trials were included in the analysis, regardless of accuracy in the GJT. We opted for this approach, since using only trials associated with correct behavioral responses would have resulted in different signal to noise ratios across learners, especially those with the highest and lowest accuracy rates. In addition, the cross-sectional study by Tanner et al. [50] compared all-trial vs. response-contingent analyses and found similar patterns of brain responses for both types of analyses. Following artifact rejection, we segmented the continuous EEG into epochs in the interval between –300 and +1200ms relative to the onset of the critical word. Epochs were averaged per condition and for each subject, and baseline-corrected relative to the 300ms pre-stimulus interval. A 30Hz low-pass digital filter was then applied to the averaged waveforms.

**Fig 1:**
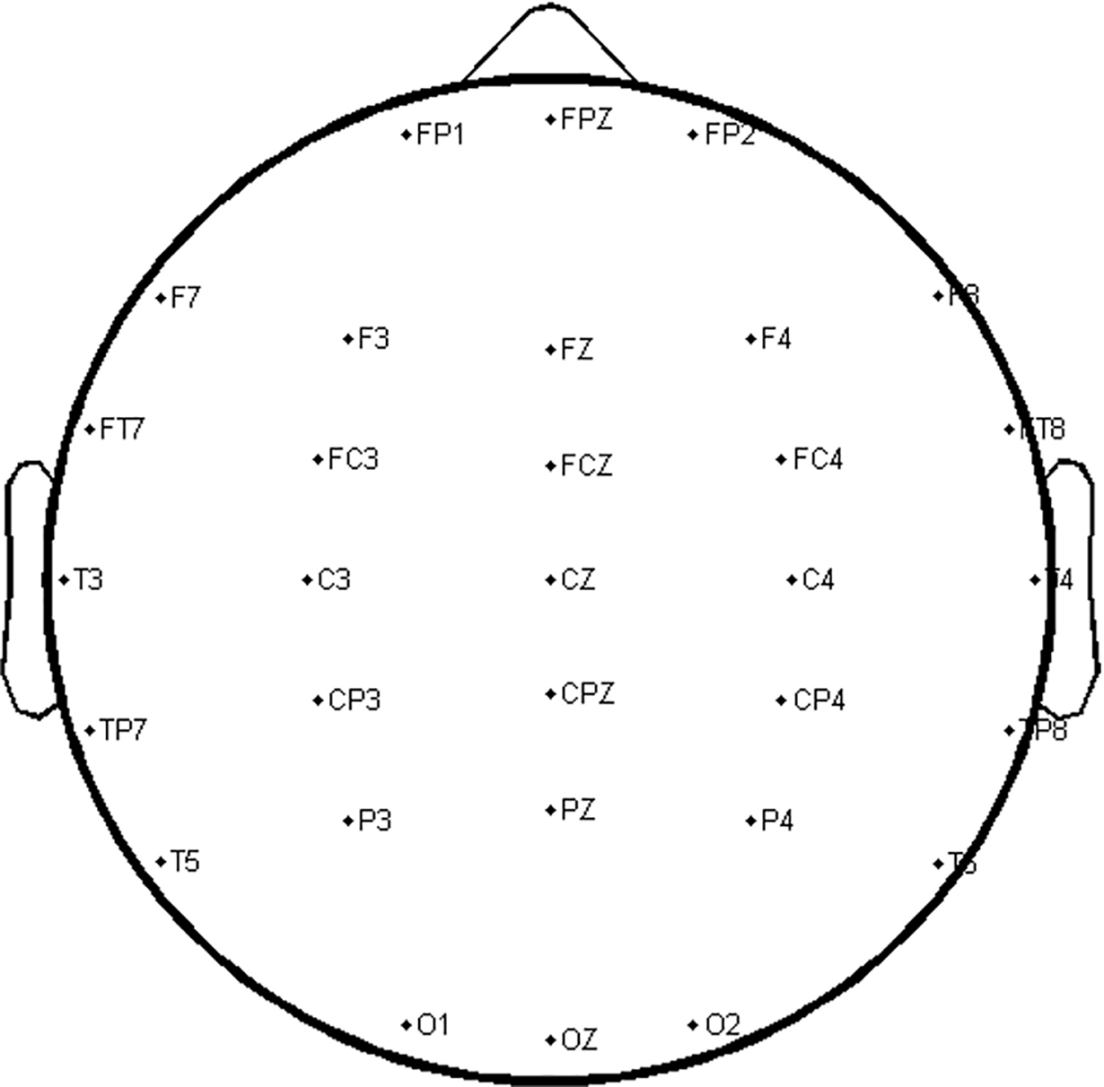
Electrode layout.

ERPs were quantified by mean amplitudes in the 250-400ms time window, which includes both the N400 [91] and the LAN [92], and the 400-900ms time window, which includes the P600 [27, 28, 92]. We computed six regions of interest (ROI) for statistical analysis: Left Anterior (FP1, F7, F3, FT7, and FC3), Right Anterior (FP2, F8, F4, FT8, and FC4), Left Posterior (TP7, CP3, T5, P3, and O1), Right Posterior (TP6, CP4, T6, P4, and O2), Midline Anterior (FPZ, FZ, and FCZ), and Midline Posterior (CPZ, PZ, and OZ). An additional Central-Posterior ROI (CP3, CPz, CP4, P3, Pz, P4, O1, Oz, O2) was calculated for correlational analyses. This region is suitable for characterizing both N400 and P600, which tend to be most evident at centro-posterior electrode sites. Fig 1 shows the electrode layout for the 32-channel system.

### Analyses

We report two main sets of analyses that focus on the question of L2 morphosyntactic development. These analyses follow the approach taken by a study by Pakulak & Neville [93] investigating the role of proficiency on syntactic processing in English monolinguals. In the first set of analyses, we conduct regression analyses from 78 learners across the whole proficiency spectrum (unlike previous studies). One advantage of this analytical approach is that it allows us to examine the individual contribution of several proficiency measures to L2 morphosyntactic development (global L2 proficiency, length of immersion in Spanish-speaking countries, and amount of Spanish instruction), including behavioral sensitivity to the properties of interest (measured via D-Prime scores). As discussed by Bowden et al. [55], little is known about the unique contribution of these measures to the development of the processing routines underlying L2 processing.

The second type of analysis uses a between-subjects design and focuses on a subset of 36 learners. Following Pakulak & Neville [93], learners were divided into two groups, Highest-Proficiency (*n* = 18) and Lowest-Proficiency (*n* = 18), based on their score in the proficiency test. That is, the 18 learners with the highest scores in the proficiency test were selected for the Highest-Proficiency group (range: 43-50, out of 50), and the 18 learners with the lowest scores were selected to create the Lowest-Proficiency group (range: 12-23, out 50). Only learners who scored above 90% in the Vocabulary and Gender Assignment Tasks were selected. Therefore, if learners are unable to detect agreement violations, we can rule out lexical problems (lack of knowledge of lexical gender, unfamiliar vocabulary) as the main explanation for it. As these two groups represent the two extremes of the L2 proficiency continuum, this approach allows us to further examine how development is modulated by L1-L2 similarity and hierarchical structure.

#### All participants analyses

For each agreement type (number, gender) and context (within-phrase, across-phrase), we calculated both D-Prime Scores [94] and ERP magnitudes. ERP magnitudes were calculated by subtracting the grammatical from the ungrammatical condition. This was done for Central-Posterior both in the 250-400ms (N400) and in the 400-900ms (P600) time windows, and for Left Anterior in the 250-400ms time window (LAN). This yielded a total of four D-Prime Score and 12 ERP Magnitude outcome variables.

A series of linear multiple regressions was carried out to examine the extent to which the learners’ overall L2 proficiency/experience accounted for their ability to discriminate between grammatical and ungrammatical sentences in the GJT. The regression model included Proficiency Test Score, Instruction, and Months Abroad as predictors, and the outcome variables were D-Prime Score (a total of four regressions). The same model was used to examine how well L2 proficiency/experience predicted the magnitude of the L2ers’ brain responses to the agreement violations. In this case, the dependent variables were (1) ERP Magnitude between 250-400ms in Left Anterior (LAN); (2) ERP Magnitude in the 250-400ms time window in Central-Posterior (N400); and (3) ERP Magnitude between 400-900ms in Central-Posterior (P600) (a total of 12 regressions). For ease of presentation, we refer to the latter outcome variables as LAN Magnitude, N400 Magnitude, and P600 Magnitude. Due to the large number of tests, we controlled for Type I error by applying a Bonferroni correction when evaluating the regression coefficients (α = .017).

#### Highest- and Lowest-Proficiency group analyses

For these analysis, a mixed-factors ANOVA was conducted with Agreement (grammatical, number violation, gender violation), Distance (within-phrase, across-phrase), Hemisphere (left, right), and Anterior-Posterior (anterior, posterior) as within-subjects factors, and Group (highest-proficiency, lowest-proficiency) as the between-subjects factor. Analyses were conducted separately for the hemispheres and the midline, which comprise different numbers of electrodes (five vs. three). For the midline regions, Anterior-Posterior was the only topographical factor in the ANOVA. The Geisser and Greenhouse correction was applied for violations of sphericity. Degrees of freedom are reported after correction [95]. A Bonferroni correction was applied for follow-up tests to control for Type I error.

## Results

### All participants analyses (*N* = 78)

Table 3 includes all of the correlations between variables related to the learners’ overall L2 proficiency (Proficiency Test Score) and experience with the L2 (Instruction, Months Abroad) and measures of behavioral and neurophysiological sensitivity to number and gender agreement (D-prime Score and ERP Magnitude, respectively). Correlations between D-Prime Scores and ERP Magnitudes (i.e. relation between behavioral and neurophysiological sensitivity) for a given agreement type and syntactic context are provided in the rightmost column of Table 3.

**Table 3:** Zero-order correlations between L2 proficiency and experiential measures (Proficiency Test Score, instruction, Months Abroad) and measures of sensitivity to agreement (D-prime Score, ERP magnitude).

	Proficiency Test Score	Instruction	Months Abroad	D-prime Score
**D-prime Score** **Number-Within**	(*r* = .621***)	(*r* = .347***)	(*r* = .356***)	
**D-prime Score** **Gender-Within**	(*r* = .594***)	(*r* = .298**)	(*r* = .369***)	
**D-prime Score** **Number-Across**	(*r* = .580***)	(*r* = .278*)	(*r* = .331**)	
**D-prime Score** **Gender-Across**	(*r* = .581***)	(*r* = .279*)	(*r* = .373***)	
**ERP magnitude (250-400ms) Number-Within Left Anterior**	(*r* = -.080)	(*r* = .006)	(*r* = .092)	(*r* = -.140)
**ERP magnitude (250-400ms) Gender-Within Left Anterior**	(*r* = .102)	(*r* = .092)	(*r* = .149)	(*r* = -.024)
**ERP magnitude (250-400ms) Number-Across Left Anterior**	(*r* = -.085)	(*r* = .032)	(*r* = -.062)	(*r* = .080)
**ERP magnitude (250-400ms) Gender-Across Left Anterior**	(*r* = .067)	(*r* = .060)	(*r* = .050)	(*r* = .041)
**ERP magnitude (250-400ms) Number-Within Posterior**	(*r* = -.130)	(*r* = -.144)	(*r* = -.054)	(*r* = -.046)
**ERP magnitude (250-400ms) Gender-Within Posterior**	(*r* = .066)	(*r* = -.130)	(*r* = .070)	(*r* = -.004)
**ERP magnitude (250-400ms) Number-Across Posterior**	(*r* = -.074)	(*r* = -.048)	(*r* = -.036)	(*r* = .058)
**ERP magnitude (250-400ms) Gender-Across Posterior**	(*r* = .106)	(*r* = -.029)	(*r* = .097)	(*r* = .146)
**ERP magnitude (400-900ms)** **Number-Within Posterior**	(*r* = .104)	(*r* = .129)	(*r* = .171)	(*r* = .386***)
**ERP magnitude (400-900ms)** **Gender-Within Posterior**	(*r* = .100)	(*r* = .035)	(*r* = .268*)	(*r* = .335**)
**ERP magnitude (400-900ms)** **Number-Across Posterior**	(*r* = .103)	(*r* = .148)	(*r* = .097)	(*r* = .472***)
**ERP magnitude (400-900ms)** **Gender-Across Posterior**	(*r* = .228*)	(*r* = .081)	(*r* = .142)	(*r* = .487***)

*p ≤ .05

**p ≤ .01

***p ≤ .001

Correlations between D-prime scores and ERP magnitudes are provided in the rightmost column. All correlations were calculated for all 78 learners. Instruction: Number of years of L2 instruction; Months Abroad: Number of months immersed in an L2-speaking country

Preliminary analyses of standardized residuals were carried out for each regression model, in order to evaluate the assumptions of regression. In all cases, the histogram of standardized residuals showed approximately normally distributed errors (skewness < 1). This was also the case for the P-P plot of standardized residuals, which showed points very close to the regression line. The scatterplot of standardized predicted values suggested that the assumptions of homogeneity of variance and linearity were also met. Importantly, the assumption of no perfect multicollinearity between the predictors was met (Proficiency Score, Tolerance = .58, *VIF* = 1.74; Months Abroad, Tolerance = .63, *VIF* = 1.59; Instruction, Tolerance = .69, *VIF* = 1.45). One outlier was removed when regressing LAN Magnitude for Across-Phrase Gender on the predictor variables.

For each participant included in our analyses, Appendix A in S1 File provides mean amplitudes for each of the six experimental conditions. Mean amplitudes are provided for each electrode and for each the two time windows under investigation (250-400, 400-900ms). Also included are accuracy rates for each of the six conditions in the GJT (percentage correct), alongside information regarding the participant’s age of acquisition, proficiency group, Proficiency Test Score, Months Abroad, and Instruction. Appendix A in S2 File provides the minimum and maximum values of the standardized residuals and the Durbin-Watson statistic for each regression. As can be seen, the final data contained no outliers and the assumption of independent errors was met.

#### Behavioral measures (D-Prime Scores)

**Fig 2:**
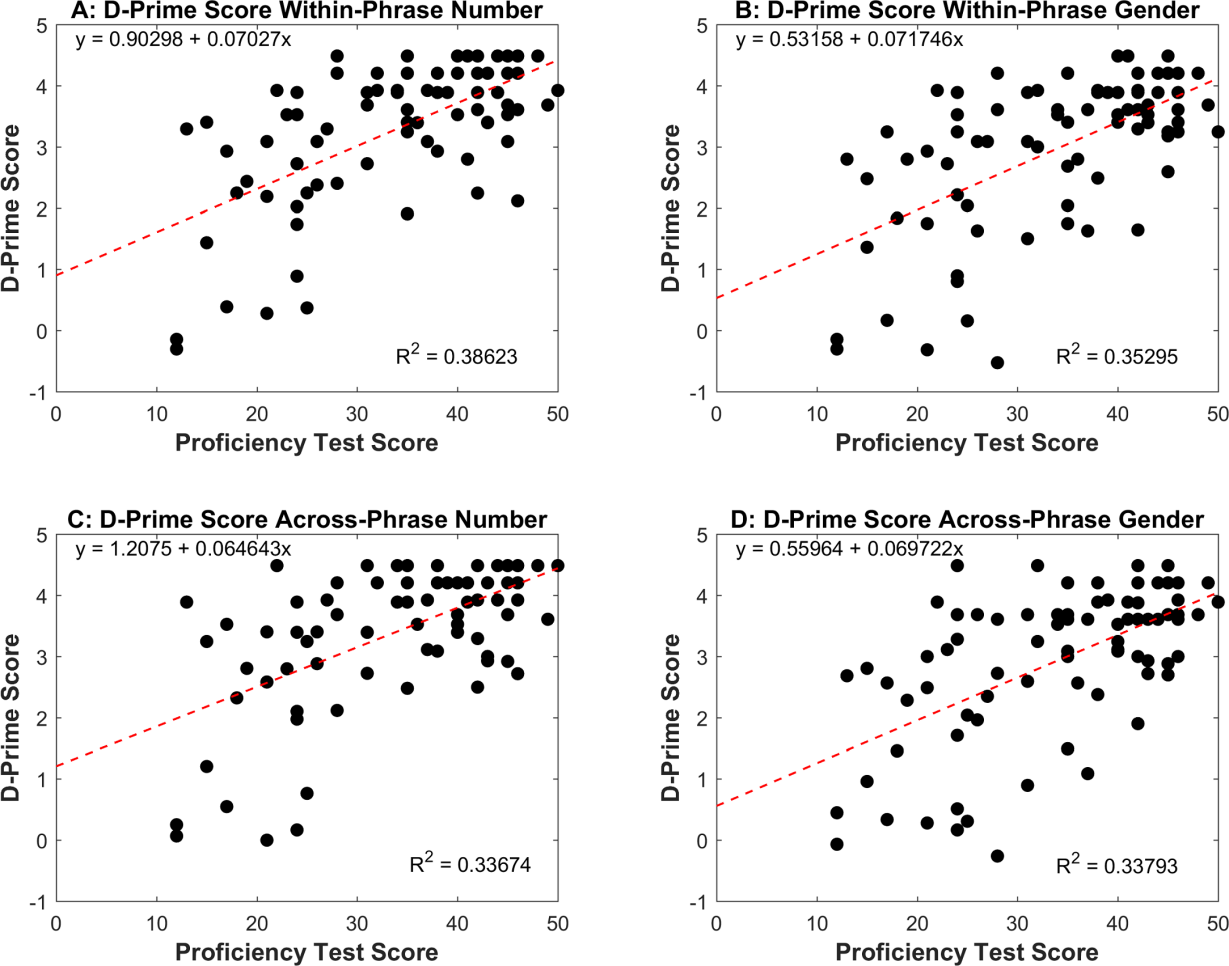
Relationship between the learners’ overall proficiency in the L2 (Proficiency Test Score) and accuracy in the grammaticality judgment task (D-Prime Score) according to agreement type. (A) Correlation between Proficiency Test Score and D-Prime Score for Within-Phrase Number. (B) Correlation between Proficiency Test Score and D-Prime Score for Within-Phrase Gender. (C) Correlation between Proficiency Test Score and D-Prime Score for Across-Phrase Number. (D) Correlation between Proficiency Test Score and D-Prime Score for Across-Phrase Gender.

As shown in Table 3, each D-prime Score variable positively correlated with Proficiency Test Score, Instruction, and Months Abroad, suggesting that, as proficiency and experience with the L2 increase, learners tend to be more sensitive to both number and gender agreement dependencies, both local and nonlocal. The model including Proficiency Test Score, Instruction, and Months Abroad accounted for a significant amount of the variance in each D-prime Score variable and, in all cases, the effect size was medium (Within-Phrase Number: *F*(3, 74) = 15.58, *p* < .001, *R*^*2*^ = .387, *adjusted R*^*2*^ = .362; Across-Phrase Number: *F*(3, 74) = 12.575, *p* < .001, *R*^*2*^ = .338, *adjusted R*^*2*^ = .311; Within-Phrase Gender: *F*(3, 74) = 13.526, *p* < .001, *R*^*2*^ = .354, *adjusted R*^*2*^ = .328; Across-Phrase Gender: *F*(3, 74) = 12.777, *p* < .001, *R*^*2*^ = .341, *adjusted R*^*2*^ = .315). Evaluation of the regression coefficients showed that, in all cases, only Proficiency Test Score made a unique contribution towards explaining variability in D-prime Scores (for Within-Phrase Number, Proficiency Test Score: *β* = .614, *t*(77) = 5.117, *p* < .001, accounting for approximately 22% of the variance, *sr*^*2*^ = .22; for Across-Phrase Number, Proficiency Test Score: *β* = .601, *t*(77) = 4.817, *p* < .001, accounting for approximately 21% of the variance, *sr*^*2*^ = .21; for Within-Phrase Gender, Proficiency Test Score: *β* = .585, *t*(77) = 4.746, *p* < .001, accounting for approximately 20% of the variance, *sr*^*2*^ = .20; for Across-Phrase Gender, Proficiency Test Score: *β* = .570, *t*(77) = 4.584, *p* < .001, accounting for approximately 19% of the variance, *sr*^*2*^ = .19). Fig 2 shows the relationship between Proficiency Test Score and each of the D-Prime Score variables.

Thus, the above regressions reveal a clear picture. The model including measures of overall L2 proficiency (Proficiency Test Score) and experience (Instruction, Months Abroad) accounts for L2 learners’ ability to discriminate between correct sentences and sentences with number/gender agreement errors, realized either within or across the phrase. However, after controlling for the effects of overall L2 proficiency, Instruction and Months Abroad do not significantly contribute to the regression. Although both Instruction and Months Abroad significantly correlated with the learners’ D-Prime Score, their relationship with behavioral accuracy appears to be mediated by overall L2 proficiency (despite the lack of perfect multicollinearity between the predictors).

#### ERP magnitudes

As shown in Table 3, very few significant correlations emerged between the magnitude of the learners’ brain responses to agreement errors and their overall L2 proficiency (Proficiency Test Score) and experience with the L2 (Instruction, Months Abroad). Interestingly, the only significant correlations that emerged involved gender agreement. This is consistent with the possibility that the mechanisms involved in the processing of novel L2 properties develop as proficiency/experience with the L2 increases.

Number agreement. The regression model including Proficiency Test Score, Instruction, and Months Abroad did not explain a significant proportion of the variance in any of the ERP Magnitude measures calculated for number. In addition, none of the coefficients for the individual predictors reached significance. This was true for both within- and across-phrase number agreement, in both time windows under investigation (250-400ms, 400-900ms), and in the two regions examined (Left Anterior, Central-Posterior). These analyses are, therefore, not reported here. Given the size of the correlations between ERP magnitudes for the number variables and the predictors, this is unsurprising (see Table 3).

**Fig 3:**
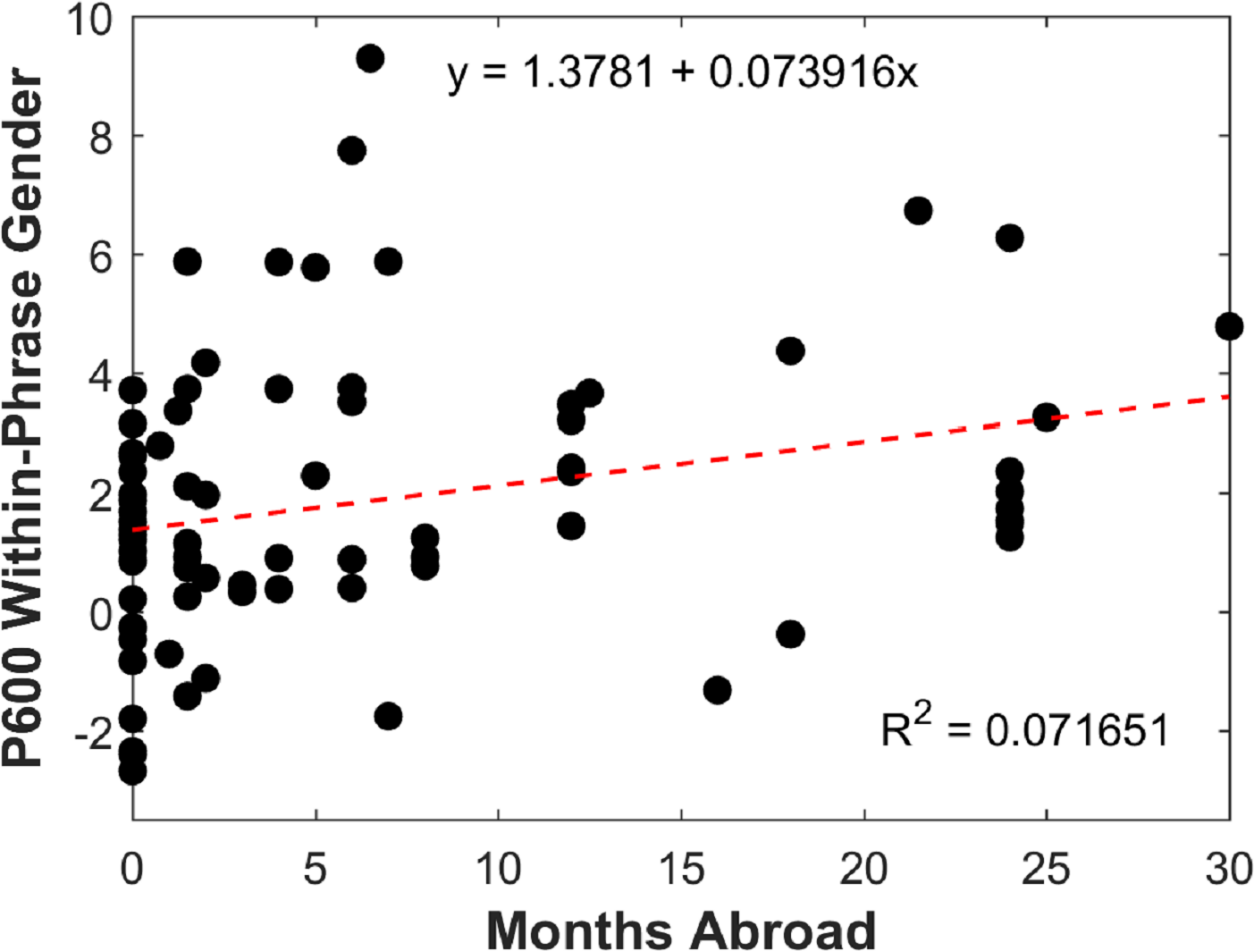
Relationship between length of immersion in Spanish-speaking countries (Months Abroad) and P600 magnitude for gender violations realized within the phrase (calculated for the Central-Posterior region).

Gender agreement. The only relevant relationship revealed by the regressions involved P600 Magnitude for Within-Phrase Gender. The model including Proficiency Test Score, Instruction, and Months Abroad accounted for a marginal proportion of the variability in P600 Magnitude for Within-Phrase Gender, *F*(3, 74) = 2.22, *p* = .093, *R*^*2*^ = .08, *adjusted R*^*2*^ = .045, an effect of small size. Most importantly, evaluation of the regression coefficients showed that Months Abroad made a unique contribution towards explaining variability in P600 Magnitude for Within-Phrase Gender (Months Abroad: *β* = .34, *t*(77) = 2.41, *p* = .018), accounting for approximately 7.2% of the variance (*sr*^*2*^ = .072). This effect became marginal (*p* = .054) after applying the Bonferroni correction. The sign of the regression coefficient suggests a positive relation between the predictor and the outcome variable, such that a one unit increase in Months Abroad (i.e. one month of immersion) resulted in an increase of .094μV in P600 amplitude. This is shown in Fig 3.

The above regression analyses suggest that overall L2 proficiency and experience do not significantly predict the development of the mechanisms underlying the processing of number agreement. With respect to gender, immersion in L2-speaking countries (as measured by Months Abroad) predicted the magnitude of the learner’s P600 to gender errors realized within the phrase, although the amount of variance explained was small and the effect became marginal after correcting for Type I error. Although Proficiency Test Score significantly correlated with P600 Magnitude for Across-Phrase Gender, its individual contribution after accounting for the effects of Instruction and Months Abroad was not different from zero (Proficiency Test Score: *β* = .24, *t*(77) = 1.631, *p* > .1).

#### Correlations between D-Prime Scores and ERP magnitudes

**Fig 4:**
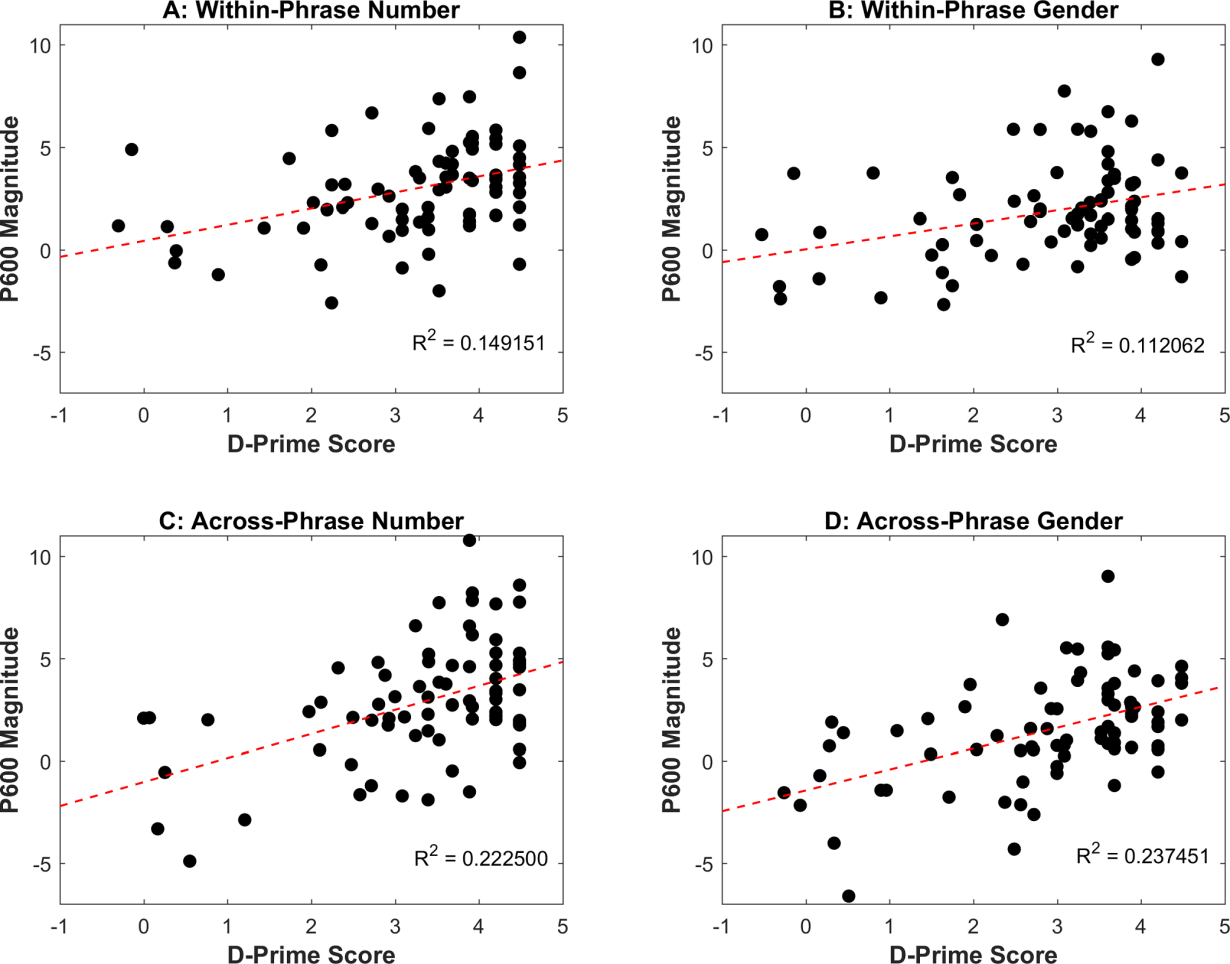
Relationship between the learners’ accuracy in the grammaticality judgment task (D-Prime Score) and P600 magnitude for each agreement type and syntactic context. (A) Correlation between D-Prime Score and P600 Magnitude for Within-Phrase Number. (B) Correlation between D-Prime Score and P600 Magnitude for Within-Phrase Gender. (C) Correlation between D-Prime Score and P600 Magnitude for Across-Phrase Number. (D) Correlation between D-Prime Score and P600 Magnitude for Across-Phrase Gender.

Additional correlational analyses were conducted between D-Prime Scores and ERP Magnitudes, to examine the relationship between behavioral and electrophysiological sensitivity to the target properties. As shown in Table 3, for each of the four agreement variables (Within-Phrase Number, Across-Phrase Number, Within-Phrase Gender, Across-Phrase Gender) there was a moderate positive correlation between D-Prime Score and P600 Magnitude, suggesting that the L2 learners with the highest accuracy in the grammaticality judgment task also tended to show the largest brain responses (i.e. positivities) to the agreement violations. These correlations are shown in Fig 4. This is consistent with previous claims that behavioral sensitivity to the target property (as measured by D-Prime Score) might be a better predictor of L2 neurocognitive development than overall L2 proficiency [50, 71], especially if we bear in mind that measures of overall L2 proficiency/experience were not found to consistently predict the magnitude of the learners’ brain responses. D-prime Score did not significantly correlate with either LAN or N400 magnitude.

#### Additional analyses: Response Magnitude Index, Response Dominance Index

One unexpected finding from the above regression analyses is that Proficiency Test Score predicted the learners’ ability to detect all four types of agreement errors (as measured by D-Prime Score), but not the magnitude of their brain responses to the violations. We hypothesized that this might be related to individual (qualitative) differences with respect to processing strategy. Recall that Tanner et al. [50] found that L2 learners at similar levels of proficiency elicited either an N400 or a P600 for the same subject-verb violations, which they interpreted as evidence that learners progress through qualitatively different processing stages (from lexically-based to rule-based) and that there is variability with respect to how fast they transition from one stage to the next. Subsequent work by Tanner and colleagues reports similar patterns for highly proficient learners [96] and even native speakers [45]. It is thus possible that, in our study, learners at similar levels of proficiency detected the agreement violations, but elicited qualitatively different brain responses to them, which might have obscured a potential relationship between the L2ers’ Proficiency Test Score and ERP Magnitude. To evaluate this possibility, we calculated both the Response Magnitude Index (RMI) and the Response Dominance Index (RDI) for each agreement type and context (a total of eight measures), following Tanner et al. [96]. RMI is a measure of the absolute magnitude of the L2ers’ brain responses to the violations, regardless of polarity. In turn, RDI is a measure of the L2ers’ dominance with respect to whether they elicited an N400 or a P600 to the violations, regardless of magnitude. We then regressed the L2ers’ RMI and RDI on the predictors, but none of these analyses revealed any significant effects. As discussed in Alemán Bañón et al. [68], it might be the case that factors other than individual differences with respect to processing strategy account for the lack of a relationship between these measures. It is also possible that inherent differences between the N400 and P600 components (e.g. the magnitude of the P600 tends to be larger than that of the N400; the P600 is usually longer-lasting) make it difficult to combine the two measures into a more general measure of sensitivity to the violations.

#### Summary of all participants analyses

Regression analyses revealed that the strongest effects of proficiency were mainly observed in D-Prime Scores, a measure of behavioral sensitivity to the target properties. Our results also revealed a strong relationship between behavioral sensitivity to the target property (as measured by D-Prime Scores) and the magnitude of the learners’ brain responses to the violations. Finally, measures of overall L2 proficiency and experience did not consistently predict ERP magnitude to the agreement errors, although length of immersion in Spanish-speaking countries (i.e. Months Abroad) was found to modulate P600 Magnitude for Within-Phrase Gender. Subsequent analyses ruled out the possibility that the lack of a relationship between overall L2 proficiency and ERP Magnitude was driven by individual differences with respect to processing strategy.

### Highest- and Lowest-Proficiency group analyses

In the following analyses we compare the 18 Highest-Proficiency and the 18 Lowest-Proficiency learners in the sample, to better understand how morphosyntactic development is impacted by hierarchical structure and L1-L2 similarity. This classification was carried out based upon the learners’ performance on the proficiency test, following a similar procedure by Pakulak & Neville [93]. These two groups represent the upper and lower ends of the proficiency spectrum, where differences in development are most likely to be observed. For each group, Table 4 provides information regarding the learners’ scores in the proficiency test (“Proficiency Test Score”), amount of Spanish instruction (“Instruction”), and length of immersion in Spanish-speaking countries (“Months Abroad”). Information regarding the learners’ age of acquisition and age at the time of testing is also included.

**Table 4:** Proficiency and age information about the Highest- and Lowest-Proficiency groups.

	Highest (*n* = 18)	Lowest (*n* = 18)
Proficiency Test Score	range: 43-50; mean: 45.5; SD: 1.95	range: 12-24; mean: 20; SD: 4
Instruction (years)	range: 2-9; mean: 4.34; SD: 1.80	range: 0-4; mean: 2.10; SD: 1.07
Months Abroad	range: 0-24; mean: 13.11; SD: 8.20	range: 0-6; mean: 1.11; SD: 1.8
Age at Testing	range: 21-41; mean: 27; SD: 5.40	range: 18-24; mean: 21; SD: 1.32
Age of Acquisition	range: 10-22; mean: 15; SD: 3.50	range: 11-20; mean: 15; SD: 2.62

Instruction: Number of years of L2 instruction; Months Abroad: Number of Months immersed in an L2-speaking country

A one-way ANOVA revealed that the Highest- and Lowest-Proficiency groups did not differ with respect to age of acquisition, *F* (1, 34) < 1. As for age at the time of testing, the ANOVA showed differences between the two groups, *F* (1, 34) = 22.43, *p* < .001, driven by the fact that the Highest-Proficiency learners were older than the Lowest-Proficiency learners when the testing took place (similar to Rossi et al. [63]). The Highest-Proficiency learners had spent more time in Spanish-speaking countries on average, even after controlling for age at the time of testing, *F* (1, 34) = 11.88, *p* < .01. Along the same lines, amount of Spanish instruction was higher for the Highest-Proficiency group, although the difference became marginal after controlling for age at the time of testing, *F* (1, 34) = 4.01, *p* = .053. It is unsurprising that there is some association between these measures of proficiency.

#### Behavioral results: GJT

Mean accuracy scores for both the within-phrase conditions (Table 2: conditions 1-3) and the across-phrase conditions (Table 2: conditions 4-6) are provided in Table 5 below. Mean accuracy scores for the Spanish native speakers reported in Alemán Bañón et al. [37] are also included for comparison.

**Table 5:** Accuracy scores (percentage of correct responses) in the grammaticality judgment task and D-Prime Scores for the Lowest- and Highest-proficiency groups.

	Within-Phrase					Across-Phrase				
	GRA	*NUM	*GEN	d’NUM	d’GEN	GRA	*NUM	*GEN	d’NUM	d’GEN
**Lowest** **(n = 18)**	84 (26)	80 (23)	76 (26)	2.35 (1.27)	2.15 (1.35)	84 (25)	82 (22)	73 (26)	2.48 (1.41)	2.15 (1.41)
**Highest** **(n = 18)**	96 (4)	97 (6)	96 (5)	3.83 (.61)	2.91 (.94)	96 (6)	97 (8)	94 (8)	3.87 (.61)	3.62 (.56)
**Native** **(n = 24)**	97 (3)	97 (4)	98 (2)	3.93 (.57)	3.98 (.45)	98 (3)	98 (3)	98 (3)	3.93 (.49)	4 (.37)

*GEN: Gender violation; GRA: Grammatical

*NUM: Number violation; d’GEN: D-prime gender; d’NUM: D-prime number. Standard Deviations are provided in parentheses.

As can be seen, even the Lowest-Proficiency group performed well above chance with both grammatical sentences and agreement violations. As expected, differences in accuracy can be observed between the two learner groups, with the Highest-Proficiency learners showing similar accuracy scores to the native speakers, and the Lowest-Proficiency learners performing worse than both the native speakers and the Highest-Proficiency learners. Accuracy values were submitted to a mixed-factors analysis of variance with Distance (Within-phrase, Across-phrase) and Agreement (Grammatical, Number, Gender) as the within-subjects factors and Proficiency (Highest-Proficiency, Lowest-Proficiency) as the between-subjects factor. A Bonferroni correction was applied for all post-hoc tests, in order to correct for Type I error.

The only significant result revealed by the omnibus ANOVA was a main effect of Proficiency (*F*(1,34) = 13.95, *p* = .001), driven by the fact that the Lowest-Proficiency learners were less accurate than the Highest-Proficiency learners across the board. These findings are consistent with the results of the above regression analyses (*N* = 78), which show that overall L2 Proficiency (Proficiency Test Score) reliably predicts behavioral accuracy with the target properties. Interestingly, the distance and agreement manipulations did not impact accuracy scores within either of the two proficiency groups.

#### ERP results

Time window between 250-400ms. Results of the omnibus ANOVA conducted for the 250-400ms time window are summarized in Appendix A in S3 File. As shown in Appendix A in S3 File, the omnibus ANOVA revealed an Agreement by Proficiency interaction, suggesting that the overall effects of the agreement manipulation affected the two proficiency groups differently. Since the present study is mainly concerned with morphosyntactic development as a function of proficiency, we decided to examine each proficiency group individually.

**Fig 5:**
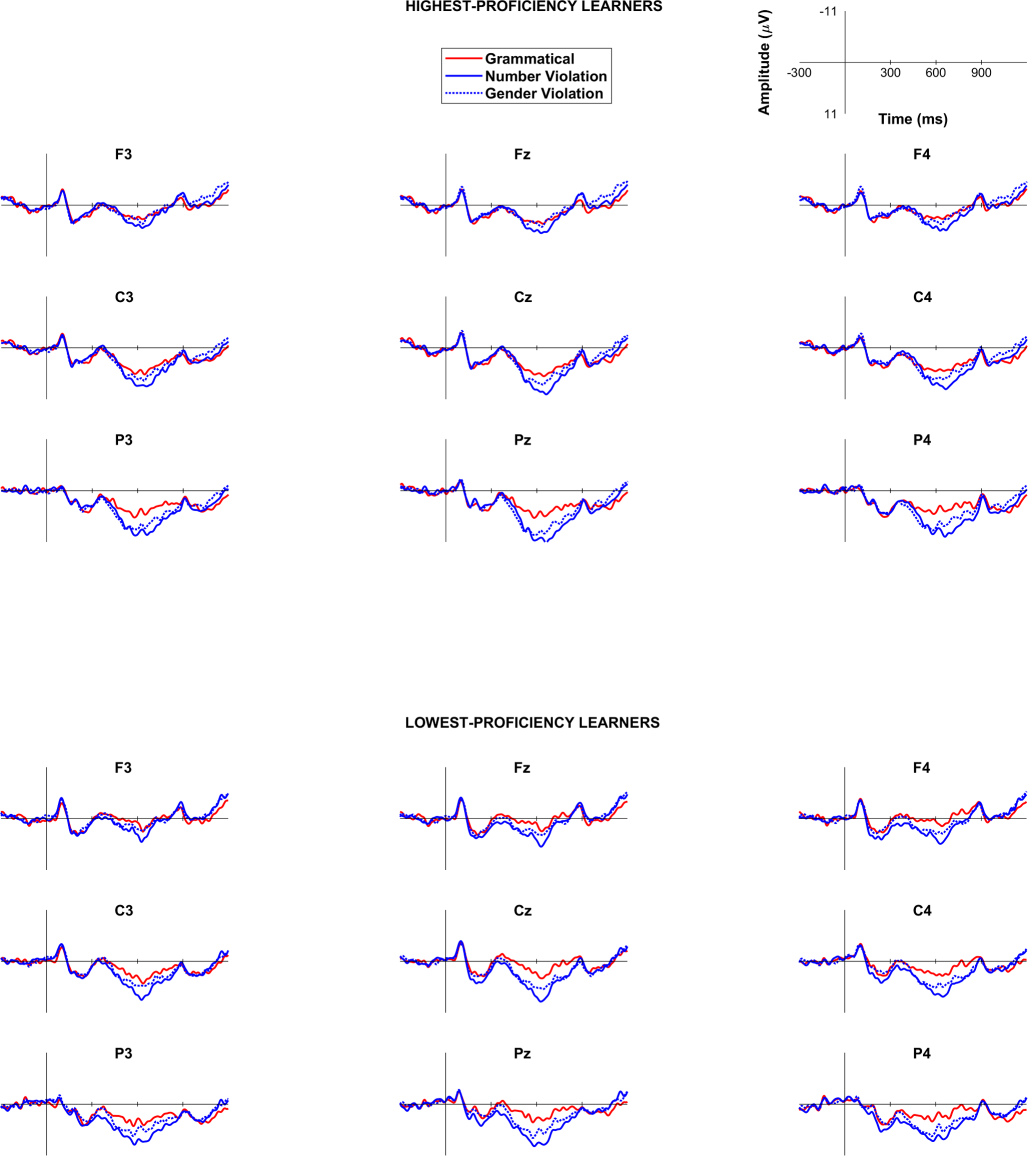
Grand average ERPs for the within-phrase agreement conditions in the Highest- and Lowest-Proficiency groups: Grammatical, number violation, and gender violation. ERPs are plotted for a representative electrode within each region of interest.

**Fig 6:**
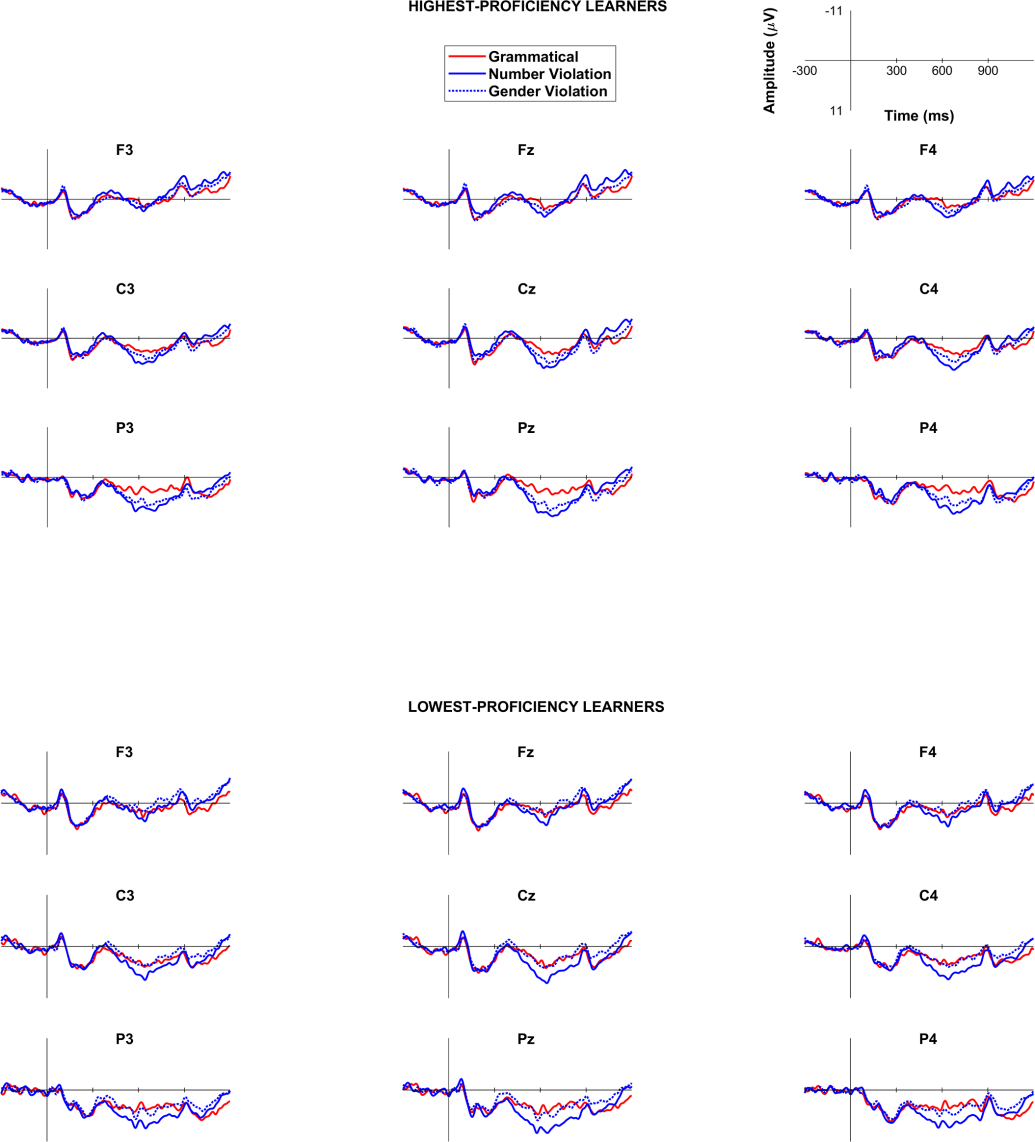
Grand average ERPs for the across-phrase agreement conditions in the Highest- and Lowest-Proficiency groups: Grammatical, number violation, and gender violation. ERPs are plotted for a representative electrode within each region of interest.

Table 6 summarizes the results of the ANOVAs that we conducted separately for each proficiency group. As can be seen, in the Highest-Proficiency group, analyses revealed an Agreement by Hemisphere by Anterior-Posterior interaction and a main effect of Agreement. Follow-up tests were conducted within each level of Anterior-Posterior, in order to examine the nature of the interaction. No effects emerged in the posterior regions. In the anterior regions, however, analyses revealed that number violations yielded more negative waveforms than grammatical sentences and gender violations in the left hemisphere, although neither effect remained significant after correcting for Type I error (grammatical vs. number: *F*(1,17) = 8.88, *p unadjusted* = .008; *p adjusted* > 1; number vs. gender violations: *F*(1,17) = 9.74, *p unadjusted* = .006, *p adjusted* > 1). This trend can be seen in Fig 5 (within-phrase agreement) and especially in Fig 6 (across-phrase agreement). In the Lowest-Proficiency group, analyses revealed a marginal Agreement by Hemisphere interaction (see Table 6). Follow-up tests revealed that number violations yielded more positive waveforms than gender violations in the right hemisphere, which might be indicative of an earlier onset of the P600 for number, although this effect also did not remain significant after Bonferroni-correcting the *p* values, *F*(1,17) = 8.03, *p unadjusted* < .05, *p adjusted* > 1.

**Table 6:** Results of the separate ANOVAs conducted in the 250-400ms time window for the Highest- and Lowest-Proficiency groups. Only significant and marginal results are reported. Where applicable, degrees of freedom were adjusted using the Greenhouse-Geisser correction.

LATERAL REGIONS: Effects	Highest	Lowest
distance x agreement x hemisphere x anterior	*—*	—
agreement x hemisphere x anterior	*F*(2,34) = 11.10***	—
distance x hemisphere x anterior	—	—
distance x agreement x anterior	—	—
agreement x anterior	*—*	—
distance x anterior	—	—
distance x agreement x hemisphere	—	—
agreement x hemisphere	—	*F*(2,34) = 4.06^
distance x hemisphere	*—*	—
distance x agreement	**—**	—
agreement	*F*(2,34) = 3.30^	—
distance	**—**	—
**MIDLINE REGIONS: Effects**		
distance x agreement x anterior	—	—
agreement x anterior	—	—
distance x anterior	—	—
distance x agreement	—	—
agreement	—	—
distance	—	—

*p ≤ .05

**p ≤ .01

***p ≤ .001

^*p* ≤ .1

The analyses for the midline regions (Appendix A in S3 File) also revealed an Agreement by Proficiency interaction. Thus, we analyzed the two proficiency groups separately. Follow-up tests revealed no significant effects of agreement or distance in either group.

Time window between 400-900ms. Results of the omnibus ANOVA for the analyses conducted in the 400-900ms time window are summarized in Appendix A in S3 File. As can be seen, results revealed a Distance by Agreement by Hemisphere by Anterior-Posterior by Proficiency interaction. Visual inspection of the interaction plots and examination of the cell means suggested that the two proficiency groups yielded different patterns of results for both the distance and agreement manipulations. As shown in Fig 5 and Fig 6, the Highest-Proficiency learners yielded more positive waveforms for both number and gender violations, relative to grammatical sentences. This effect appears larger for number than gender and mainly shows a posterior distribution. In addition, the effect appears similar for violations realized within and across the phrase. Finally, the waveforms for all within-phrase conditions appear more positive than their across-phrase counterparts, an effect that is evident across the scalp.

As shown in Fig 5 and Fig 6, the Lowest-Proficiency learners yielded more positive waveforms for number violations relative to grammatical sentences both within and across the phrase. This effect shows a posterior distribution. Gender violations, in contrast, were slightly more positive than grammatical sentences (especially in the within-phrase configuration), although the effect does not appear robust (especially across the phrase) and is only apparent in the posterior portion of the right hemisphere.

In order to better characterize these group differences, we decided to analyze the two proficiency groups separately. Table 7 summarizes the results of the ANOVAs that we conducted separately for each proficiency group. In the Highest-Proficiency group, post-hoc tests revealed a four-way interaction between Distance, Agreement, Hemisphere, and Anterior-Posterior. Analyses also revealed an Agreement by Hemisphere by Anterior-Posterior interaction, an Agreement by Anterior-Posterior interaction, an Agreement by Hemisphere interaction, a main effect of Agreement, and a main effect of Distance. Follow-up tests were conducted within each level of Distance, in order to examine the nature of the Distance by Agreement by Hemisphere by Anterior-Posterior interaction. In the within-phrase configuration, results revealed an Agreement by Anterior-Posterior interaction (*F*(2,34) = 17.94, *p* < .001) and a main effect of Agreement (*F*(2,34) = 10.17, *p* < .01). Follow-up tests revealed that both number and gender violations yielded more positive waveforms than grammatical sentences (number: (*F*(1,17) = 39.67, *p* < .001; gender: (*F*(2,34) = 19.55, *p* < .01), mainly in the posterior regions. Effects for number and gender differed numerically, but not statistically. In the across-phrase configuration, results revealed an Agreement by Hemisphere by Anterior-Posterior interaction (*F*(2,34) = 6.81, *p* < .05), an Agreement by Anterior-Posterior interaction (*F*(2,34) = 13.05, *p* < .001), an Agreement by Hemisphere interaction (*F*(1.37,23.36) = 7.61, *p* < .05), and a main effect of Agreement (*F*(2,34) = 5.16, *p* < .05). Follow-up tests for the three-way interaction (by Anterior-Posterior) revealed no effects of agreement in the anterior regions. In the posterior regions, analyses revealed a main effect of Agreement (*F*(1.71,25.71) = 18.22, *p* < .001), driven by the fact that both number and gender violations yielded more positive waveforms than grammatical sentences (number: (*F*(1,17) = 25.37, *p* < .01; gender: (*F*(2,34) = 32.18, *p* < .001). As was the case in the within-phrase context, effects for number and gender did not significantly differ. Finally, the main effect of Distance was driven by the fact that within-phrase agreement yielded more positive waveforms than across-phrase agreement overall.

**Table 7:** Results of the separate ANOVAs conducted in the 400-900ms time window for the Highest- and Lowest-Proficiency groups. Only significant and marginal results are reported. Where applicable, degrees of freedom were adjusted using the Greenhouse-Geisser correction.

LATERAL REGIONS: Effects	Highest	Lowest
distance x agreement x hemisphere x anterior	*F*(2,34) = 4.37*	—
agreement x hemisphere x anterior	*F*(2,34) = 4.17*	—
distance x hemisphere x anterior	—	—
distance x agreement x anterior	—	—
agreement x anterior	*F*(2,34) = 20.54***	*F*(2,34) = 7.12**
distance x anterior	—	—
distance x agreement x hemisphere	—	—
agreement x hemisphere	*F*(2,34) = 4.76*	*F*(2,34) = 9.91***
distance x hemisphere	*—*	—
distance x agreement	**—**	—
Agreement	*F*(2,34) = 11.51***	*F*(2,34) = 7.85**
Distance	*F*(1,17) = 7.43*	—
**MIDLINE REGIONS: Effects**		
distance x agreement x anterior	—	—
agreement x anterior	*F*(2,34) = 19.27***	*F*(2,34) = 8.15**
distance x anterior	—	—
distance x agreement	—	*F*(2,34) = 3.33^
Agreement	*F*(2,34) = 17.72***	*F*(2,34) = 7.57**
Distance	*F*(1,17) = 11.25**	—

*p ≤ .05

**p ≤ .01

***p ≤ .001

^*p* ≤ .1

In the Lowest-Proficiency group, analyses revealed an Agreement by Hemisphere interaction, an Agreement by Anterior-Posterior interaction, and a main effect of Agreement. Follow-up tests to the Agreement by Anterior-Posterior interaction revealed a main effect of Agreement in the posterior portion of the EEG cap only (*F*(2,34) = 10.42, *p* < .01), driven by the fact that number violations yielded more positive waveforms than grammatical sentences (*F*(2,34) = 19.63, *p* < .01). Number violations were also more positive than gender violations, although the effect was only significant before correcting for Type I error, *F*(1,17) = 8.38, *p unadjusted* = .01; *p adjusted* > 1. Follow-up tests for the Agreement by Hemisphere interaction suggested that the effects for number violations showed a right hemisphere bias (main effect of Agreement in right hemisphere, *F*(2,34) = 9.62, *p* < .01; number: *F*(1,17) = 18.13, *p* < .05).

For consistency, analyses in the midline regions were also conducted separately for the Highest- and Lowest-proficiency learners, and they showed a similar pattern of results to the hemispheres.

#### Summary of Highest- and Lowest-Proficiency group analyses

With respect to the distance manipulation, the two proficiency groups showed different profiles. While the Highest-Proficiency learners showed more positive waveforms for all within-phrase conditions relative to their across-phrase counterparts in the 400-900ms time window (similar to the native speakers in Alemán Bañón et al. [37]), the Lowest-Proficiency group appeared to be insensitive to whether the agreeing elements belonged in the same phrase or in different phrases. With respect to the agreement manipulation, results also revealed different profiles for the two proficiency groups. For number violations at both levels of structural distance, both groups yielded a posteriorly distributed P600 between 400-900ms. In contrast, only the Highest-Proficiency learners showed a P600 for gender violations, both within and across the phrase. In the Lowest-Proficiency group, gender violations were numerically more positive than grammatical sentences, but the effect was not significant at either level of structural distance.

In the Highest-Proficiency group, number violations also yielded more negative waveforms than both grammatical sentences and gender violations in the 250-400ms time window, an effect that showed a left anterior bias. This effect is compatible with the Left Anterior Negativity, although it should be pointed out that it did not remain robust after correcting for Type I error. In addition, it did not emerge in the native speaker group for either number or gender violations, at either level of structural distance [37].

## Discussion

We discuss these findings with respect to the specific research questions that motivate our study. Our first question concerns the role of proficiency in L2 morphosyntactic development. Our results revealed a strong role for proficiency, although differences emerged between behavioral and electrophysiological measures of sensitivity to the target properties. When the whole proficiency spectrum was examined via regression analyses, overall L2 proficiency (operationalized as performance on a comprehensive L2 grammar test) was found to reliably predict the learners’ accuracy in the GJT with both number and gender agreement, realized both within and across the phrase. In contrast, proficiency did not reliably predict the magnitude of the learners’ brain responses (i.e. P600) to agreement violations. A significant positive correlation did emerge between overall L2 proficiency and P600 Magnitude for across-phrase gender, but regression analyses showed that the individual contribution of this predictor was not significant after controlling for other (also non-significant) factors (i.e. amount of L2 instruction and length of immersion in L2-speaking countries). Effects of proficiency on the ERP data did emerge when comparing the two ends of the proficiency spectrum via between-groups analyses (e.g. [93]). Here, we found that the Highest-Proficiency learners were qualitatively similar to the Spanish controls reported in Alemán Bañón et al. [37]. In contrast, the Lowest-Proficiency learners differed from both the Highest-Proficiency group and the native speakers in two important ways. First, they only showed sensitivity to number (i.e. P600), the property that is present in their L1. In addition, although they were sensitive to number violations realized both within and across the phrase, they were unaffected by the structural distance manipulation, consistent with the possibility that, at lower levels of proficiency, L2ers might not be sensitive to subtle differences in hierarchical structure in real-time comprehension (we return to both points later in the discussion).

It remains unclear to us why proficiency effects on the ERP data only emerged in the between-groups analysis. One of the explanations that we considered concerns individual differences with respect to processing strategy. Based on the results by Tanner et al. [50], we hypothesized that learners at similar levels of proficiency might show brain responses of different polarity to the agreement errors (N400 vs. P600), which might have obscured a potential relationship between overall L2 proficiency and ERP magnitude. However, our follow-up analyses using the magnitude of the learners’ brain responses to the violations irrespective of polarity (i.e. Response Magnitude Index) did not support this hypothesis (see also [68]). It might simply be the case that subtle differences in how learners perform on a comprehensive test of L2 vocabulary and grammar do not correspond to subtle changes in brain sensitivity to the specific morphosyntactic properties that we examined.

A related question that we addressed concerns the individual contribution of different proficiency-related factors to L2 morphosyntactic development. We approached this question by examining both overall L2 proficiency and measures of experience with the L2 (amount of L2 instruction, length of immersion in L2-speaking countries). Our results suggest that overall L2 proficiency was the more reliable predictor of morphosyntactic development at the behavioral level, since it was the only factor that made a significant contribution towards explaining variability in all D-Prime Scores. Although both experiential variables were positively correlated with all D-Prime Scores, regression analyses revealed that this relationship was mediated by overall L2 proficiency. This is not surprising, since some association between overall L2 proficiency, amount of instruction, and length of immersion is expected, despite the lack of perfect multicollinearity between the three predictors. With respect to the ERP data, length of immersion in L2-speaking countries made an individual contribution towards explaining variability in P600 Magnitude for within-phrase gender. It is therefore possible that immersion in an L2-speaking environment helps to consolidate the mechanisms associated with the processing of novel morphosyntactic features, although strong conclusions are precluded, since this effect became marginal after correcting for Type I error and it was not found for across-phrase gender. Unlike Caffarra et al. [73], length of immersion in our study was unrelated to LAN magnitude.

Our results also revealed a significant relationship between the learners’ accuracy with the target properties (as measured by D-Prime Scores in the GJT) and their electrophysiological sensitivity to them, similar to previous studies (e.g. [49, 50, 71]). Recall that the meta-analysis by Caffarra et al. [73] found that proficiency was the most reliable predictor of P600 Magnitude for morphosyntactic errors, although their analysis did not tease apart global L2 proficiency from behavioral sensitivity to the target property. The results of the present study suggest that it is the latter measure that most reliably predicts electrophysiological sensitivity to L2 morphosyntactic dependencies. In this respect, our results contrast with a previous study by Tokowicz & MacWhinney [85]. The authors examined number and gender agreement in L1-English L2-Spanish learners at a low/intermediate level of proficiency and found electrophysiological sensitivity to gender errors (i.e. P600) despite chance performance in the GJT. Tokowicz & MacWhinney argue that implicit sensitivity to grammatical properties can develop before the learners acquire explicit knowledge of those properties. The significant correlations between D-Prime Scores and P600 Magnitude that we found for all agreement types, however, suggest that behavioral and electrophysiological sensitivity develop together.

An interesting observation is that we did not find N400 effects for any type of agreement error at any point in development. Under the assumption that L2 morphosyntactic processing relies on lexically-based strategies (indexed by the N400) at lower levels of proficiency (as predicted by certain L2 processing models; [16, 20–22]; see also [49, 50]) the prediction is that there should be an inverse relationship between the learners’ proficiency (with the target property or overall L2 proficiency) and N400 magnitude, such that learners at lower levels of proficiency would elicit larger N400 effects. Another possibility is that the learners’ proficiency would correlate with Response Dominance Index, a measure of the learners’ dominance with respect to whether they elicited an N400 or a P600 to the agreement errors. However, our regression analyses revealed no significant relationships between any of these variables. In addition, the between-groups analyses comparing the two extremes of the proficiency spectrum also failed to reveal N400 effects for any of the target properties in the Lowest-Proficiency group. One possibility is that reliance on lexically-based strategies takes place at even lower levels of proficiency (i.e. novice learners), as was the case in the study by Osterhout et al. [49]. In our study, the Lowest-Proficiency learners had received an average of two years of Spanish instruction at the time of testing, suggesting that they might have been past the purported N400-stage, despite being very low-proficiency and despite having been tested on a novel instantiation of number agreement (i.e. on the adjective). We point out, however, that two longitudinal studies from our own lab looking at novice learners also failed to reveal N400 effects for either number or gender agreement violations at any stage of development. In addition, some studies have reported N400 effects for gender violations in L2 learners at an advanced level of proficiency (e.g. [48]). It is therefore unclear exactly at what point in development (and for which property) L2 learners might be more reliant on lexical heuristics as opposed to rule-based knowledge.

An interesting finding from our between-groups analyses is that, in the 250-400ms time window, both types of number violations were more negative than grammatical sentences in the Left Anterior region, an effect that is consistent with the LAN. Although this effect did not remain robust after adjusting α for multiple comparisons, the fact that this negativity only emerged in the Highest-Proficiency group is consistent with the possibility that L2 morphosyntactic processing becomes increasingly automatized at higher levels of proficiency/experience (at least for properties that exist in the L1) (e.g. [63, 65]), which is partly consistent with processing models such as the Declarative/Procedural Model and with Steinhauer et al.’s proposed developmental trajectory [16]. However, the fact that the L1 Spanish controls did not show this effect for any agreement type calls for caution when interpreting the LAN as an index of native-like morphosyntactic processing.

The second question that we investigated concerns the role of L1-L2 similarity on morphosyntactic development. The results from our between-groups analyses clearly suggest that the L1 modulates development, but does not constrain it. At the lowest levels of proficiency, learners only showed electrophysiological sensitivity to number, the property that is instantiated in their L1, even though their accuracy with number and gender in the GJT did not significantly differ. In addition, this sensitivity was in the form of a P600, the same component elicited by the L1-Spanish controls. At the highest level of proficiency, however, learners showed equally robust P600 effects for both number and gender. Interestingly, the larger P600 that we reported for number than gender in the advanced learners in Alemán Bañón et al. [67] is no longer present in this Highest-Proficiency group, suggesting that transfer effects might not be a permanent phenomenon.

The fact that the Lowest-Proficiency learners showed evidence of grammatical processing for properties that exist in their L1 is consistent with theoretical models which assign a privileged role to the L1 at early stages of development (Full Transfer Full Access Hypothesis, [14, 15]; Interpretability Hypothesis, [17]). Although, a priori, our results are not consistent with models which assume that L2 processing initially relies on lexically-based strategies, we point out that even less proficient learners might be necessary to fully evaluate this possibility. As for the Highest-Proficiency learners, the fact that they showed qualitatively native-like processing for gender is only consistent with models which assume that development for novel properties to native-like levels is possible (e.g. Full Transfer Full Access Hypothesis).

Interestingly, facilitation for number in the Lowest-Proficiency group emerged despite the fact that agreement was examined on adjectives, a syntactic context where English does not mark any type of agreement (e.g. [71]). This challenges claims that adult L2ers cannot process novel instantiations of a shared feature in a native-like manner, either at low (e.g. [85]) or even advanced levels of proficiency (e.g. [48]). We point out that, although our data did reveal facilitation for number (present in L1), they also provide evidence for its development, as suggested by (1) the significant correlations between overall L2 proficiency and D-Prime Scores for number and (2) the significant correlations between D-Prime Scores and P600 Magnitude for number.

Our last research question concerns how linguistic factors such as structural distance (i.e. hierarchical structure) impact L2 morphosyntactic development. In the Lowest-Proficiency group, we found that learners could detect (number) agreement violations realized both within and across the phrase, but sensitivity was not modulated by structural distance. In contrast, the Highest-Proficiency learners were affected by the structural distance manipulation similarly to the native controls (i.e. more positive waveforms for all within-phrase conditions, relative to their across-phrase counterparts). We can think of at least two interpretations for these effects. First, it is possible that, in the course of online comprehension, less proficient learners establish morphosyntactic dependencies in a more linear fashion. That is, at this stage of development, learners might be able to check agreement between nouns and adjectives (at least, for number) without necessarily relying on fine-grained abstract syntax. However, once they reach a high level of proficiency, learners rely more on hierarchical syntactic representations, similar to native speakers. This would still provide evidence that L2 processing is not permanently confined to local domains (contra the Shallow Structure Hypothesis) and that it develops to native-like levels as a function of proficiency.

Alternatively, these results might reveal proficiency-based differences in learners’ ability to generate predictions regarding the syntactic category of the critical word. The adverb *muy* “very” in the within-phrase conditions made it very likely that the following word would be an adjective, whereas the copula *es* “is” in the across-phrase conditions did not allow for as strong a prediction regarding the syntactic category of the following word. In other words, *muy* acted as a stronger predictive cue than *es*. One possibility is that, upon encountering the adverb *muy* in the within-phrase conditions, both the native speakers and the Highest-Proficiency learners were better able to anticipate the following adjective than in the across-phrase conditions. In turn, this might have impacted the waveforms for all within-phrase conditions relative to their across-phrase counterparts (regardless of grammaticality, since the category of the critical word was equally predictable across the three levels of grammaticality). In contrast, at lower levels of proficiency, processing might not be sufficiently fast or efficient for learners to be able to generate syntactic predictions, which would explain the lack of a difference between the within- and across-phrase conditions (see [97] for evidence of syntactic prediction-related positivities in native speakers of English, which were absent in adult L2 learners of English).

Under the assumption that our results are related to prediction generation, they would indicate that the ability to engage in anticipatory processing develops as a function of proficiency. In fact, a recent study by Dussias et al. [98] provides evidence that proficiency impacts anticipatory processing in adult L2ers. In their study, only the highest proficiency learners were able to use morphosyntactic cues predictively. Future ERP studies should examine how proficiency modulates adult L2 learners’ ability to generate structural predictions online.

## Conclusion

The present study reveals a strong role for proficiency in L2 morphosyntactic development, although differences emerged between behavioral and electrophysiological measures of sensitivity. We found that global L2 proficiency reliably predicted the learners’ ability to detect both number and gender violations, regardless of the structural distance between the agreeing elements. With respect to the development of the neurocognitive mechanisms recruited in morphosyntactic processing, our study also revealed a role for proficiency, but only when contrasting the two extremes of the proficiency spectrum. Our results also reveal a relation between the learners’ ability to detect agreement violations in an explicit judgment task and the size of their brain responses (i.e. P600), suggesting that both types of sensitivity might develop together. Importantly, we found that linguistic factors such as L1-L2 similarity and hierarchical structure modulate development, but do not constrain it. While at lower levels of proficiency learners are only sensitive to properties that exist in their L1 and do not show sensitivity to differences in hierarchical structure, at the highest levels of proficiency, learners show sensitivity to all properties of the L2 and are able to posit hierarchical syntactic representations online. The difference between these two groups with respect to the hierarchical structure manipulation suggests that sensitivity to certain linguistic factors, such as structural distance or syntactic predictability, emerges as overall proficiency level develops.

## Supporting information

S1 File Worksheet. Mean amplitudes, accuracy rates, and proficiency-related information (proficiency group, proficiency test score, months abroad, instruction).
(XLSX)

S2 File Table. Standardized residuals (minimum and maximum) and Durbin-Watson statistic for each regression analysis.
(DOCX)

S3 File Table. Results of the omnibus ANOVA conducted for the 250-400ms and 400-900ms time windows for the between-groups analyses (Highest- vs. Lowest-Proficiency).
(DOCX)
