# Supplementary material for: Using event-related potentials to track morphosyntactic development in second language learners: The processing of number and gender agreement in Spanish
Source: PLoS One. 2018 Jul 27;13(7):e0200791. doi: 10.1371/journal.pone.0200791 (PMC6063416; doi:10.1371/journal.pone.0200791)
Supplement: S2 File — Standardized residuals (minimum and maximum) and Durbin-Watson statistic for each regression analysis. (DOCX) [file pone.0200791.s002.docx]

**Appendix B. Standardized residuals (minimum and maximum) and Durbin‑Watson statistic for each regression analysis.**

|  | **Std. Residual**  **Minimum** | **Std. Residual**  **Maximum** | **Durbin‑Watson** |
| --- | --- | --- | --- |
| **D‑prime Score Number‑Within** | -2.504 | 1.783 | 2.31 |
| **D‑prime Score Gender‑Within** | -2.961 | 1.794 | 2.33 |
| **D‑prime Score Number‑Across** | -2.701 | 1.962 | 2.28 |
| **D‑prime Score Gender‑Across** | -2.632 | 2.224 | 2.36 |
| **ERP (250‑400ms) Number‑Within LEFT ANTERIOR** | -2.500 | 2.339 | 2.15 |
| **ERP (250‑400ms) Gender‑Within LEFT ANTERIOR** | -2.621 | 2.023 | 2.33 |
| **ERP (250‑400ms) Number‑Across LEFT ANTERIOR** | -2.210 | 2.633 | 2.02 |
| **ERP (250‑400ms) Gender‑Across LEFT ANTERIOR** | -2.680 | 2.559 | 1.78 |
| **ERP (250‑400ms) Number‑Within POSTERIOR** | -2.745 | 3.045 | 2.43 |
| **ERP (250‑400ms) Gender‑Within POSTERIOR** | -1.879 | 2.510 | 2.46 |
| **ERP (250‑400ms) Number‑Across POSTERIOR** | -2.730 | 2.306 | 1.60 |
| **ERP (250‑400ms) Gender‑Across POSTERIOR** | -2.593 | 2.522 | 1.93 |
| **ERP (400‑900ms) Number Within POSTERIOR** | -2.440 | 3.127 | 2.04 |
| **ERP (400‑900ms) Gender Within POSTERIOR** | -1.809 | 3.242 | 2.10 |
| **ERP (400‑900ms) Number Across POSTERIOR** | -2.565 | 2.736 | 2.11 |
| **ERP (400‑900ms) Gender Across POSTERIOR** | -2.887 | 2.890 | 2.35 |

*All values are reported for regressions including 78 learners, with the exception of ERP Magnitude (250‑400ms) Gender Agreement LEFT ANTERIOR, where one outlier was removed.*
