# Supplementary material for: Using event-related potentials to track morphosyntactic development in second language learners: The processing of number and gender agreement in Spanish
Source: PLoS One. 2018 Jul 27;13(7):e0200791. doi: 10.1371/journal.pone.0200791 (PMC6063416; doi:10.1371/journal.pone.0200791)
Supplement: S3 File — Results of the omnibus ANOVA conducted for the 250-400ms and 400-900ms time windows for the between-groups analyses (Highest- vs. Lowest-Proficiency). (DOCX) [file pone.0200791.s003.docx]

**Appendix C. Results of the omnibus ANOVA for the between-subjects analyses. Results are reported for the two time windows of interest (250‑400ms and 400‑900ms). Only significant results are reported. Where applicable, degrees of freedom were adjusted using the Greenhouse-Geiser correction.**

| **LATERAL REGIONS: Effects** | **250-400 ms** | **400‑900 ms** |
| --- | --- | --- |
| distance x agreement x hemisphere x anterior x proficiency | *---* | *F*(2, 68) = 4.051* |
| distance x agreement x hemisphere x anterior | *---* | **---** |
| agreement x hemisphere x anterior x proficiency | *---* | **---** |
| agreement x hemisphere x anterior | *F*(2, 68) = 9.859*** | *F*(2, 68) = 5.985** |
| distance x hemisphere x anterior x proficiency | **---** | *F*(1, 34) = 3.985^ |
| distance x hemisphere x anterior | **---** | **---** |
| distance x agreement x anterior x proficiency | **---** | **---** |
| distance x agreement x anterior | **---** | **---** |
| agreement x anterior x proficiency | **---** | **---** |
| agreement x anterior | *---* | *F*(2, 68) = 24.035*** |
| distance x anterior x proficiency | *---* | **---** |
| distance x anterior | **---** | **---** |
| distance x agreement x hemisphere x proficiency | *---* | **---** |
| distance x agreement x hemisphere | *F*(2, 68) = 3.906* | **---** |
| agreement x hemisphere x proficiency | **---** | **---** |
| agreement x hemisphere | *F*(2, 68) = 5.456** | *F*(2, 68) = 13.00*** |
| distance x hemisphere x proficiency | **---** | **---** |
| distance x hemisphere | *---* | *F*(1, 34) = 4.984* |
| distance x agreement x proficiency | *---* | *---* |
| distance x agreement | **---** | *F*(2, 68) = 2.586^ |
| agreement x proficiency | *F*(2, 68) = 4.954** | **---** |
| agreement | *---* | *F*(2, 68) = 16.727*** |
| distance x proficiency | *---* | *---* |
| distance | *F*(1, 34) = 3.384^ | *---* |
| proficiency | *---* | *---* |
| **MIDLINE REGIONS: Effects** |  |  |
| distance x agreement x anterior x proficiency | **---** | **---** |
| distance x agreement x anterior | **---** | **---** |
| agreement x anterior x proficiency | **---** | **---** |
| agreement x anterior | *F*(2, 68) = 3.174* | *F*(2, 68) = 24.866*** |
| distance x anterior x proficiency | *---* | **---** |
| distance x anterior | **---** | **---** |
| distance x agreement x proficiency | *---* | *---* |
| distance x agreement | *F*(2, 68) = 2.657^ | *F*(2, 68) = 2.652^ |
| agreement x proficiency | *F*(2, 68) = 3.927* | **---** |
| agreement | *---* | *F*(2, 68) = 19.313*** |
| distance x proficiency | **---** | *F*(1, 34) = 4.695* |
| distance | *---* | **---** |
| proficiency | *---* | *---* |
